# Multimodal interactions drive chromatin phase separation and compaction

**DOI:** 10.1073/pnas.2308858120

**Published:** 2023-12-04

**Authors:** Tina Ukmar-Godec, Maria-Sol Cima-Omori, Zhadyra Yerkesh, Karthik Eswara, Taekyung Yu, Reshma Ramesh, Gwladys Riviere, Alain Ibanez de Opakua, Wolfgang Fischle, Markus Zweckstetter

**Affiliations:** ^a^German Center for Neurodegenerative Diseases, Translational Structural Biology, Göttingen 37075, Germany; ^b^Bioscience Program, Biological and Environmental Science and Engineering Division, Laboratory of Chromatin Biochemistry, King Abdullah University of Science and Technology, Thuwal 23955, Saudi Arabia; ^c^Department of NMR-based Structural Biology, Max Planck Institute for Multidisciplinary Sciences, Göttingen 37077, Germany

**Keywords:** Heterochromatin Protein 1α (HP1α), H3K9-trimethylation, phase separation, chromatin compaction

## Abstract

Gene silencing is intimately connected to DNA condensation and the formation of transcriptionally inactive heterochromatin by Heterochromatin Protein 1α (HP1α). Because heterochromatin foci are dynamic and HP1α can promote liquid–liquid phase separation, HP1α-mediated phase separation has been proposed as a mechanism of chromatin compaction. The molecular basis of HP1α-driven phase separation and chromatin compaction and the associated regulation by trimethylation of lysine 9 in histone 3 (H3K9me3), which is the hallmark of constitutive heterochromatin, is however largely unknown. Using a combination of chromatin compaction and phase separation assays, site-directed mutagenesis, and NMR-based interaction analysis, we show that human HP1α can compact chromatin in the absence of liquid–liquid phase separation. We further demonstrate that H3K9-trimethylation promotes compaction of chromatin arrays through multimodal interactions. The results provide molecular insights into HP1α-mediated chromatin compaction and thus into the role of human HP1α in the regulation of gene silencing.

## Significance

A correct balance between gene silencing and gene activation is essential for normal cellular function. It is established that this balance involves the reversible formation of compact, transcriptionally inactive heterochromatin and, according to recent hypotheses, may also require phase separation of heterochromatin. However, the molecular mechanism governing gene silencing remains elusive. Here, we show that chromatin compaction can occur in absence of phase separation, and we elucidate the interactions driving compaction. Our results provide insights into the molecular basis of the regulation of gene silencing.

Most of the eukaryotic genome is packaged into highly condensed, transcriptionally inactive heterochromatin (1, 2). Gene silencing by heterochromatin plays essential roles during development and cell differentiation. Heterochromatin is proposed to emerge because of heterochromatin protein 1α (HP1α) spreading across large regions of the genome, compacting the underlying chromatin and recruiting other ligands (3–5). Binding of HP1α to the trimethylated lysine 9 in histone H3 (H3K9me3) is a hallmark of the establishment and maintenance of constitutive heterochromatin (6–8). Recent studies linked chromatin compaction and in turn gene silencing to biomolecular liquid–liquid phase separation based on the observation that HP1 proteins phase separate alone (9), with DNA (10, 11), and with chromatin (12–14). In addition, chromatin itself can undergo liquid–liquid phase separation in vitro and form dynamic puncta upon injection into the cell nucleus (15). However, the mechanistic basis of HP1α-driven chromatin compaction, liquid–liquid phase separation and the associated regulation by H3K9me3, are largely unclear.

**Fig. 1.**
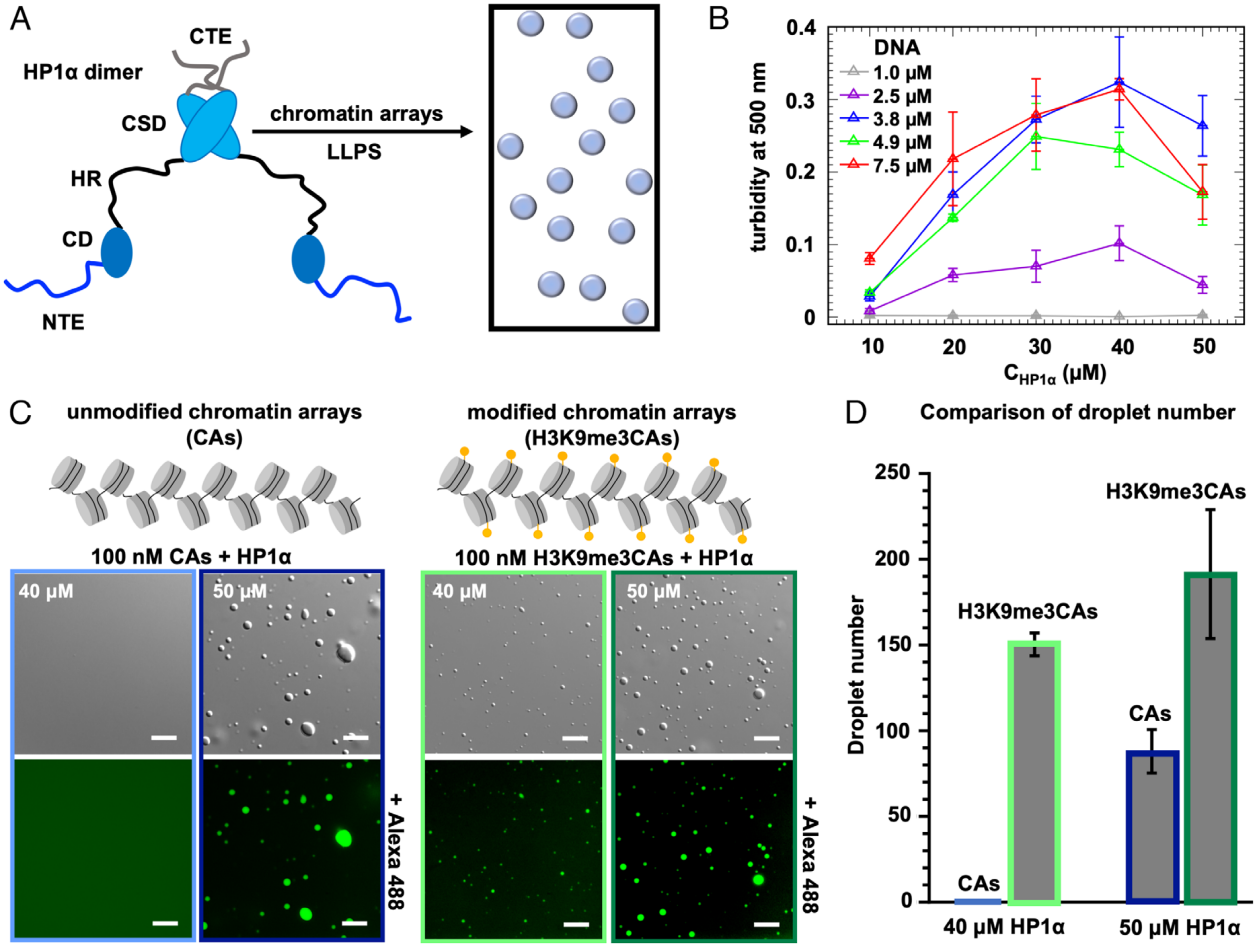
H3K9me3 promotes HP1α-mediated liquid–liquid phase separation of chromatin arrays. (*A*) Schematic representation of the HP1α dimer when it liquid–liquid phase separates with chromatin arrays. CD and CSD are the chromo and chromo-shadow domain of HP1α, respectively, NTE/CTE the N/C-terminal disordered tails, and HR the disordered hinge region. (*B*) Turbidity measurements of mixtures of HP1α with DNA for different protein (*x* axis) and DNA concentrations (indicated in the legend). Turbidity values represent the average of independent samples and the corresponding std from independent samples (n = 3). (*C*) DIC and fluorescence images showing HP1α liquid–liquid phase separation with unmodified chromatin arrays (CAs; 100 nM) (*Left*) and H3K9me3 chromatin arrays (H3K9me3CAs; 100 nM) (*Right*) at HP1α concentrations of 40 and 50 µM in 10 mM Tris-HCl, pH 7.8, 75 mM KCl, 0.5 mM EDTA, 1 mM TCEP buffer. For fluorescence imaging, unlabeled HP1α samples were mixed with Alexa 488-labeled protein (0.6 μM). (Scale bars, 10 μm.) (*D*) Quantification of HP1α/CA droplet numbers at HP1α concentrations of 40 and 50 µM using either unmodified (blue) or H3K9me3CAs (green) from panel (*C*). Error bars represent SD from three independent fluorescence images (image size: 138.33 × 103.75 μm).

HP1α contains two globular modules, termed chromo-domain (CD) and chromo-shadow domain (CSD) (Fig. 1*A*). The CD binds the H3K9me3 mark specifically but with low affinity (micromolar regime) (16–18). The CSD mediates dimerization (19, 20) and recruits nuclear proteins containing a PXVXL motif (21, 22). The CD and CSD are connected by an intrinsically disordered hinge region (HR) and flanked by disordered tails (Fig. 1*A*) (4). The flexible hinge region has been associated with DNA-binding and non-specific interaction with chromatin (23–25). Binding of DNA to the hinge region of HP1α promotes its liquid–liquid phase separation (10), consistent with the importance of intrinsically disordered regions for the formation of biomolecular condensates (26). In addition, phosphorylation of the disordered N-terminal tail (NTE) promotes liquid–liquid phase separation of human HP1α (10). Phase-separated droplets of HP1α, potentially phosphorylated or carrying other post-translational modifications (27, 28), might thus provide a means to physically sequester and compact chromatin while concurrently enabling the recruitment of repressive factors (29).

HP1α-mediated liquid–liquid phase separation of nucleosomes agrees with the dynamic properties of heterochromatin foci (29–31). In addition, liquid–liquid phase separation of chromatin carrying different histone modifications potentially enables the formation of nuclear chromatin subdomains (15). However, liquid–liquid phase separation mediated by multivalent interactions might not be the only mechanism enabling the formation of dynamic heterochromatin foci and could be complemented or replaced by bridging proteins that cross-link chromatin segments (32, 33). Insights into the mechanism of HP1α-driven chromatin compaction is further complicated by the multidomain structure of HP1α, which provides diverse opportunities for specific regulation of HP1α-driven chromatin compaction and phase separation. Here, we provide insights into the molecular basis of the compaction and liquid–liquid phase separation of chromatin by human HP1α.

## Results

### H3K9me3 Promotes HP1α-Mediated Phase Separation of Chromatin.

Methylation of H3K9 is a hallmark of heterochromatin (6–8). To investigate the influence of H3K9me3 on HP1α-mediated liquid–liquid phase separation of chromatin arrays, we determined the conditions under which human HP1α phase separates with unmodified and H3K9me3 chromatin arrays (Fig. 1 *A*, *C*, and *D*). In addition, we studied HP1α liquid–liquid phase separation with DNA and investigated the importance of the hinge region of HP1α using an HR peptide (Fig. 1*B* and *SI Appendix*, Fig. S1 *A* and *D*).

We prepared solutions of human tag-free HP1α with different concentrations of low molecular weight salmon sperm DNA (34) and monitored solution turbidity (Fig. 1*B*). With 1 μM DNA, no changes in solution turbidity were observed for increasing HP1α concentrations. Increasing the HP1α concentration in the presence of 2.5 μM DNA, however, caused a rise in turbidity. Strong concentration-dependent turbidity was also observed with 3.8, 4.9, and 7.5 μM DNA (Fig. 1*B*). We then selected the DNA concentration of 3.8 μM for subsequent HP1α liquid–liquid phase separation experiments because the DNA did not phase separate by itself at this concentration (*SI Appendix*, Fig. S1*B*). Fluorescence microscopy confirmed that DNA induced the formation of droplets (from strand-like to spherical) which are highly enriched in HP1α (*SI Appendix*, Fig. S1*C*). Droplets were also formed by the HR peptide in the presence of 1.9 μM DNA (*SI Appendix*, Fig. S1*D*).

Next, we investigated the ability of HP1α to induce phase separation of unmodified and H3K9me3 chromatin arrays (*SI Appendix*, Fig. S1 *E* and *I*). Addition of 40 μM HP1α to 100 nM of H3K9me3 chromatin arrays resulted in droplet formation, while no phase separation was detected in these conditions for unmodified chromatin arrays (Fig. 1 *C* and *D*). When increasing the HP1α concentration to 50 μM, phase separation occurred for both unmodified and H3K9me3 chromatin arrays (Fig. 1 *C* and *D*). We observed a larger number of droplets with H3K9me3 chromatin. In the case of unmodified chromatin arrays, we also observed some larger droplets, which could be the result of smaller droplets fusing into bigger droplets. Supporting a process of liquid–liquid phase separation, HP1α/chromatin droplets fused (*SI Appendix*, Fig. S1*H*) and the droplets contain both HP1α and chromatin (*SI Appendix*, Fig. S1*I*). We further note that increasing the concentration of the chromatin arrays enhanced HP1α-mediated phase separation (*SI Appendix*, Fig. S1*G*). The data show that H3K9me3, which increases the binding affinity to HP1α‘s CD to nucleosomes (16–18), promotes HP1α-mediated liquid–liquid phase separation of chromatin arrays.

### Chromatin Compaction/Association Occurs at Sub-phase Separation Concentrations.

**Fig. 2.**
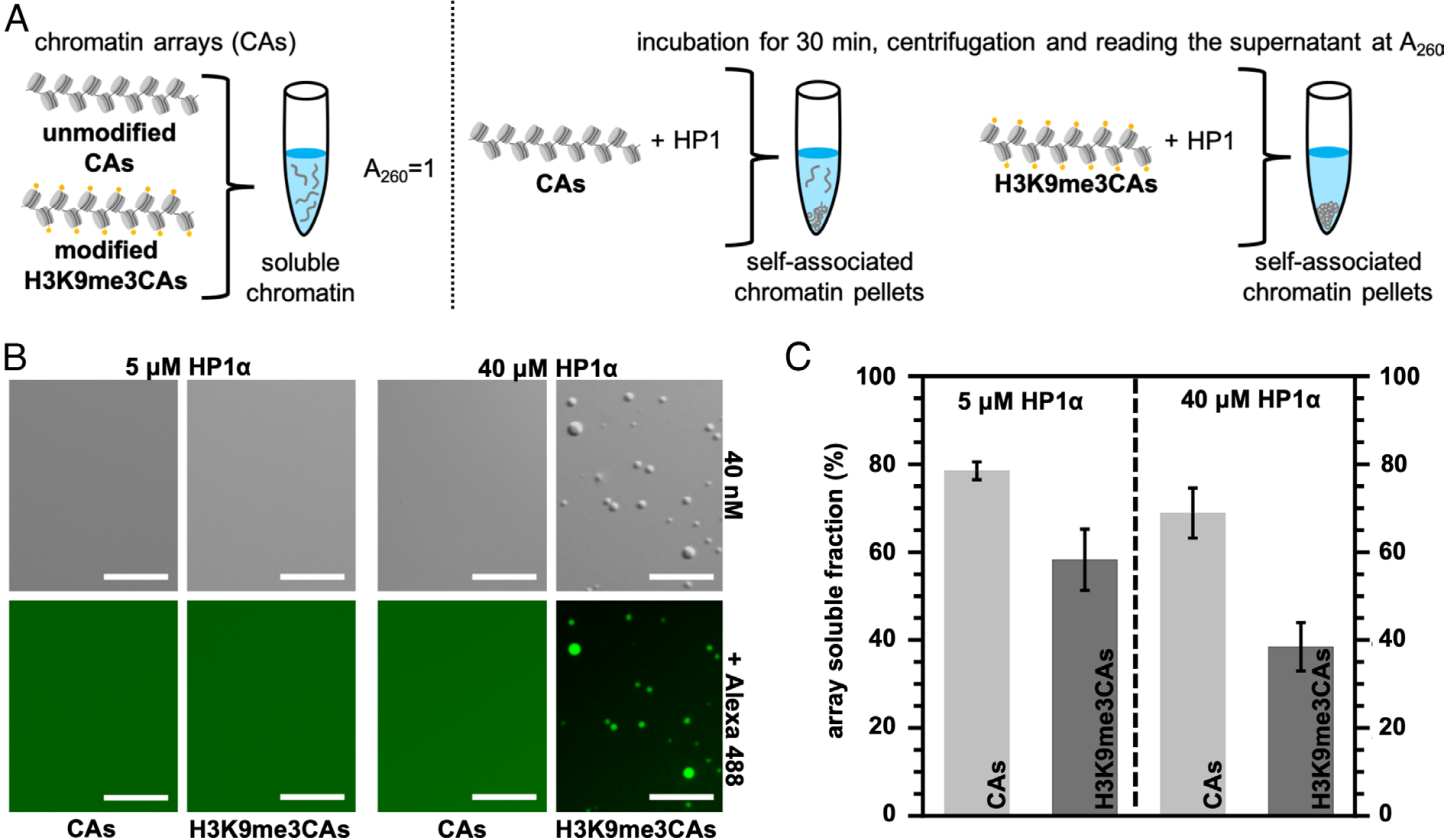
HP1α compacts chromatin at sub-phase separation concentrations. (*A*) Schematic representation of the chromatin compaction assay. (*B*) DIC images of mixtures of HP1α and chromatin arrays at different concentrations in 10 mM Tris-HCl, pH 7.8, 75 mM KCl, 0.5 mM EDTA, and 1 mM TCEP buffer. For fluorescence imaging, unlabeled HP1α samples were mixed with Alexa 488-labeled protein (0.6 μM). (*C*) Quantification of soluble chromatin upon mixing 40 nM of unmodified or H3K9me3 chromatin arrays at two different concentrations of HP1α, 5 μM and 40 μM, respectively. Error bars represent SD from independent samples (n = 3). (Scale bars, 10 μm.)

To gain insight into the importance of HP1α-mediated phase separation of chromatin for gene silencing, we studied HP1α-mediated phase separation and chromatin compaction (Fig. 2*A*). We used a chromatin association assay (12) to determine the ability of HP1α to compact chromatin arrays. The assay is based on the interaction between the protein and chromatin (inter-array self-association), in particular on quantifying the fraction of chromatin that becomes compacted/aggregated upon association with HP1α, and is in turn removed by centrifuging the sample. HP1α-bound but not compacted chromatin would not give rise to aggregates and would in turn not be eliminated by centrifugation. The assay therefore quantifies the fraction of compacted/associated chromatin. Following previously established protocols (12), we used 40 nM of unmodified chromatin arrays and HP1α concentrations of 5 μM and 40 μM (Fig. 2 *B* and *C*). To determine the concentration at which HP1α phase separates in the presence of 40 nM of unmodified and H3K9me3 chromatin, we additionally performed concentration-dependent phase separation experiments (*SI Appendix*, Fig. S2). We did not observe droplet formation at 5 μM of HP1α (Fig. 2 *B*, *Left*). At 40 μM of HP1α, only H3K9me3 chromatin arrays generated phase-separated droplets (Fig. 2 *B*, *Right*), in agreement with the phase separation-promoting effect of the methyl-mark on histone H3 (Fig. 1 *C* and *D*).

We then performed chromatin association assays in the same condition (Fig. 2*A*). In the case of unmodified chromatin, chromatin association occurred at both 5 μM and 40 μM of HP1α (Fig. 2*C*). Quantification determined the fraction of soluble chromatin as 78.5 ± 2.0% and 68.9 ± 5.7%, respectively (Fig. 2*C*). Thus, ~20% and ~30% of the unmodified chromatin was compacted at 5 μM and 40 μM of HP1α, respectively. The data show that chromatin association can occur at sub-phase separation conditions but is not very efficient with unmodified chromatin arrays. We note that previous studies showed that the *S. pombe* HP1 protein Swi6 can compact chromatin arrays already at 4 μM protein concentration (12), highlighting the differences between *S. pombe* Swi6 and human HP1α (35).

Because H3K9me3 promotes HP1α-mediated phase separation of chromatin arrays (Figs. 1*C* and 2*B*), we next tested the impact of the modification on the ability of HP1α to compact chromatin arrays. In the presence of 5 μM HP1α, 58.3 ± 7.0% of the soluble chromatin arrays were in solution (Fig. 2*C*), indicating that ~40% of H3K9me3 chromatin was compacted/associated. H3K9me3 thus promotes the association of chromatin arrays, consistent with the increased affinity of the CD of HP1α for binding the H3K9me3 mark (6). At the higher HP1α concentration of 40 μM, the fraction of soluble chromatin arrays further decreased by ~20% to 38.5 ± 5.5% (Fig. 2*C*). With H3K9me3, chromatin association is thus more sensitive to the concentration of HP1α when compared to unmodified chromatin. Because H3K9me3 but not unmodified chromatin arrays formed droplets with 40 μM but not 5 μM HP1α (Fig. 2*B*), the enhanced compaction may be associated with the phase-separated state. Alternatively, another H3K9-trimethylation-associated process that is sensitive to HP1α concentration may be present.

### Determinants of HP1α-Mediated Chromatin Compaction.

**Fig. 3.**
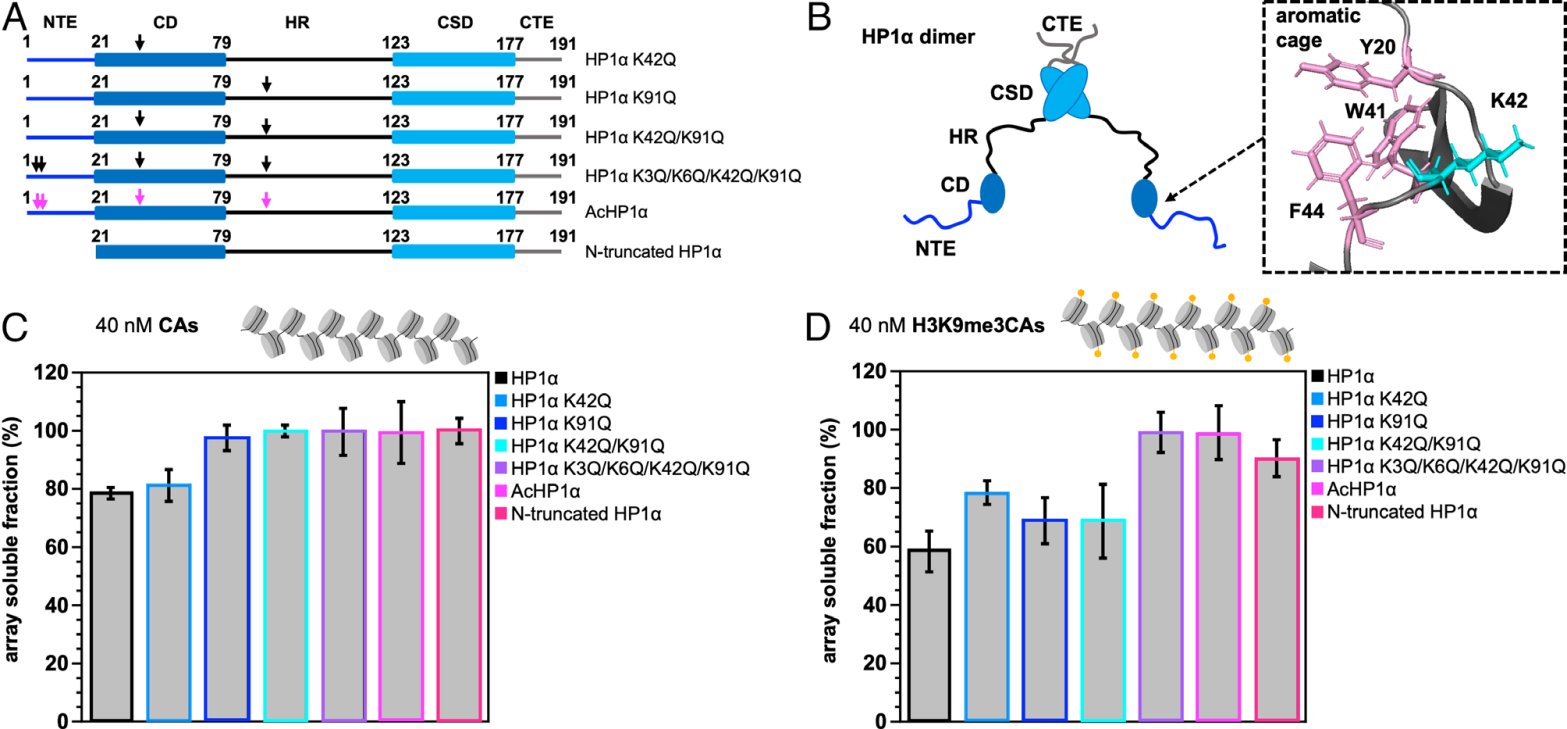
Domain contributions to HP1α-mediated chromatin compaction. (*A*) Cartoon of HP1α mutant proteins and AcHP1α. Arrows mark mutation sites. (*B*) Schematic representation of the HP1α dimer together with a detailed view of the structure of the aromatic cage formed by Y20, W41, and F44 in the CD (PDB codes: 3fdt). K42, which is the only lysine acetylated in the CD, is depicted in cyan. The structure of the CD was visualized using PyMOL version 2.1. (*C* and *D*) Soluble fractions of CAs in the presence of 5 μM of different HP1α variants reporting on the ability of HP1α to compact chromatin in a 40 nM solution of unmodified CAs (*C*) or H3K9me3 CAs (*D*); Error bars represent SD from independent samples (n = 3).

To gain insight into the molecular mechanisms determining HP1α-mediated chromatin compaction, we combined site-directed mutagenesis and chromatin association assays. To this end, we prepared an N-truncated version of HP1α that lacks the disordered N-terminal tail. Additionally, we introduced point mutations into HP1α which replace selected lysine residues by glutamine to remove positive charges at specific positions in HP1α. From the 29 lysine residues of HP1α, we selected K42 in the chromodomain and K91 in the hinge region. We also prepared a K42Q/K91Q double mutant as well as the mutant protein HP1α-K3/K6/K42/K91, in which additionally K3 and K6 in the disordered N-terminal tail were replaced by glutamine (Fig. 3*A*). The four lysine residues are located in different domains of HP1α and can be acetylated in vitro by p300/CREB (Fig. 3*A* and *SI Appendix*, Fig. S3*A*). The mutant proteins together with the acetylated and wild-type HP1α were subjected to chromatin association assays using 5 μM of protein and 40 nM chromatin arrays, i.e., conditions at which we did not detect HP1α-mediated phase separation (Fig. 2 *B*, *Left*).

For the analysis, we started with unmodified chromatin arrays (Fig. 3*C*). In the case of wild-type HP1α and the mutant protein HP1α-K42, ~20% of chromatin was compacted/associated, indicating that the K42Q mutation did not impair chromatin association. In contrast, when K91 was replaced by glutamine, either alone or in combination with K42, the amount of soluble chromatin remained unchanged (Fig. 3*C*). Lysine K91 in the hinge region thus plays an important role for HP1α-mediated compaction of unmodified chromatin arrays. Additionally, chromatin association did not occur with HP1α-K3/K6/K42/K91 or the acetylated HP1α protein (Fig. 3*C*). Acetylated HP1α was also unable or had reduced ability to phase separate with DNA or chromatin arrays (*SI Appendix*, Figs. S3 *F* and *G*, S4, and S5). Chromatin association did also not occur with the N-terminally truncated protein (Fig. 3*C*). The combined data indicate that the interaction of lysine residues in the disordered NTE and hinge region, likely with DNA, determines HP1α-mediated phase separation and chromatin association.

In the case of H3K9me3 chromatin arrays, lysine-to-glutamine mutations however displayed a different behaviour (Fig. 3*D*). Substitution of K42 by glutamine roughly halved the degree of chromatin association from ~40 to ~20%. The impact of the K42Q mutation in the CD of HP1α was thus comparable to that of the K91Q mutation in the hinge region, as well as the K42Q/K91Q double mutation (Fig. 3*D*). Additionally, the ability to compact H3K9me3 chromatin arrays was lost when all four lysine residues (K3, K6, K42, and K91) were mutated to glutamine or were acetylated (Fig. 3*D*). Notably, K42 is adjacent to the aromatic “cage,” which is formed by the three aromatic side chains of Y20, W41 and F44 (Fig. 3*B*) and mediates the specific binding to the histone H3K9me3 mark (36–38).

### Multiple HP1α Domains Contribute to Chromatin Binding.

To gain further insight into the role of HP1α domains in chromatin compaction and phase separation, we analyzed the interaction of human HP1α with DNA as well as with chromatin using NMR spectroscopy.

**Fig. 4.**
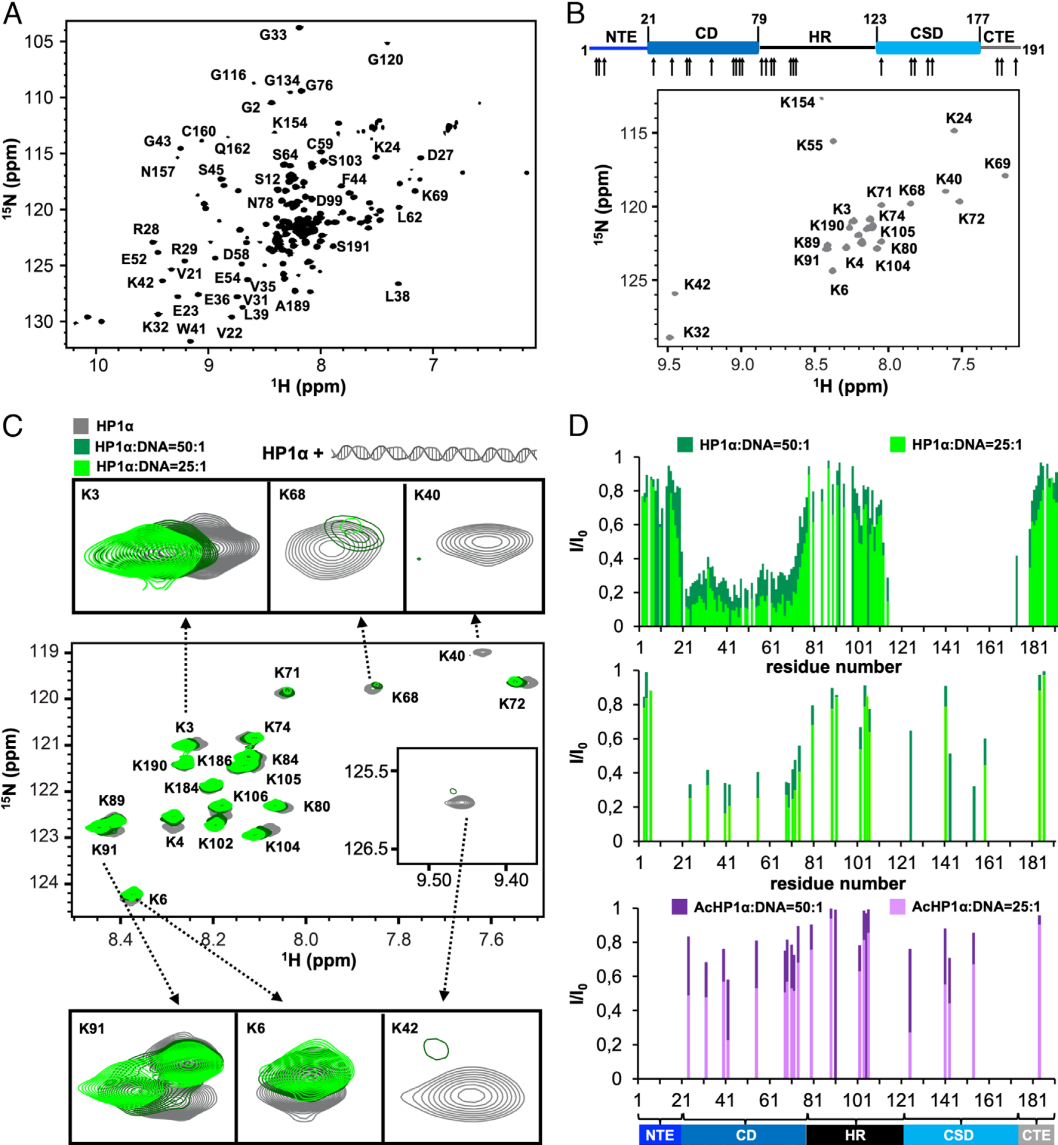
HP1α chromodomain interacts with DNA. (*A*) 2D ^1^H−^15^N TROSY-HSQC NMR spectrum of full-length HP1α. (*B*) 2D ^1^H−^15^N HSQC spectrum of ^15^N-lysine labeled HP1α; above: domain organization of HP1α with the location of its 29 lysine residues indicated by arrows. (*C*) Superposition of 2D ^1^H−^15^N HSQC spectra of ^15^N-lysine labeled HP1α (gray) without DNA or in the presence of HP1α:DNA molar ratios of 50:1 (dark green) and 25:1 (green). The cross-peaks of K3 (NTE), K6 (NTE), K40 (CD), K42 (CD), K68 (CD), and K91 (HR) are highlighted in the *Insets*. (*D*) Relative peak intensities of uniformly ^15^N-labeled HP1α (*Top*), ^15^N-lysine labeled HP1α (*Middle*), and ^15^N-lysine labeled AcHP1α (*Bottom*) at increasing HP1α:DNA molar ratios [HP1α or AcHp1α/DNA molar ratio 50:1 (dark green or purple) and 25:1 (green or lila)]. The domain organization of HP1α is shown below.

NMR assignment of full-length HP1α is complicated by the presence of both disordered and folded domains and the CSD-mediated dimerization of HP1α. We therefore used complementary triple-resonance NMR experiments to determine the protein’s backbone resonance assignment (Fig. 4*A* and *SI Appendix*, Fig. S6). Because of the unfavorable relaxation properties of the HP1α dimer, the NMR signals of most CSD residues were not detected in triple-resonance spectra and instead were transferred from those of the isolated CSD (39). To decrease signal overlap, we also produced ^15^N-lysine-labeled HP1α (Fig. 4*B*). The combined NMR peak assignment allowed us to interrogate DNA and chromatin binding of the full-length HP1α dimer.

First, we studied the interaction of HP1α with DNA. To this end, we followed the changes in NMR signal positions and intensities of HP1α residues at two different DNA concentrations using 2D ^1^H-^15^N correlation spectra (Fig. 4*C* and *SI Appendix*, Fig. S7). Addition of DNA changed both the peak positions and signal intensities of selected residues with stronger perturbations induced at higher DNA concentration (Fig. 4 *C* and *D* and *SI Appendix*, Figs. S7 and S8 *A* and *B*). In agreement with binding of the negatively charged DNA to the positively charged hinge region of HP1α, the cross-peaks of K91 and K104 were shifted and broadened (Fig. 4*C*). In addition, chemical shift perturbations were present for NTE residues (*SI Appendix*, Fig. S8 *A* and *B*) and the signal intensities of the residues within the CD strongly decreased (Fig. 4 *C* and *D* and *SI Appendix*, Fig. S7). Because the CD residues displayed predominantly peak broadening, the binding of DNA to the CD of HP1α occurs on the intermediate-to-slow NMR time scale. The data demonstrate that the NTE, the CD, and the hinge region interact with DNA and thus may contribute to HP1α-mediated chromatin compaction.

To investigate the importance of positive charges in HP1α for binding to DNA, we probed the DNA-binding of acetylated HP1α. We observed smaller DNA-induced attenuation of NMR signal intensities across the full sequence of HP1α, including the CD and the hinge region when compared to unmodified HP1α (Fig. 4*D*; compare middle and bottom panels). In addition, the extent of DNA-induced chemical shift perturbations was smaller (*SI Appendix*, Fig. S8*C*). Together, the data support the importance of electrostatic interactions between the positively charged side chains of HP1α and the negatively charged backbone phosphates of DNA.

Next, we studied the interaction of full-length HP1α with chromatin arrays. NMR titrations of HP1α in the presence of unmodified and H3K9me3 chromatin revealed predominantly signal attenuation and only small chemical shift changes (*SI Appendix*, Fig. S9). Similar to the binding to DNA, the binding of HP1α to chromatin is on the intermediate-to-slow NMR time scale. With unmodified chromatin, the signal attenuation was most apparent in the CD (*SI Appendix*, Fig. S9). Additionally, signal attenuation may have occurred in the CSD, but the small signal intensities of the CSD residues made an analysis difficult (*SI Appendix*, Fig. S9). In the case of H3K9me3 chromatin, an overall signal attenuation by ~30% occurred indicating that this fraction of HP1α no longer contributes to the NMR signal. This may arise from the stronger interaction of HP1α with H3K9me3 chromatin and the formation of high-molecular-weight HP1α/chromatin complexes. Besides this overall signal attenuation, the CD—and potentially the CSD—displayed additional signal attenuation supporting the binding of the CD to chromatin (*SI Appendix*, Fig. S9).

### The Chromodomain of HP1α Binds to DNA.

To determine whether the chromatin-induced signal broadening of the CD arises from a direct interaction with DNA, we recombinantly prepared the isolated CD of HP1α and assigned its NMR peaks (*SI Appendix*, Fig. S10). The isolated CD was subsequently titrated with DNA and the interaction was monitored by 2D ^1^H-^15^N correlation spectra (*SI Appendix*, Fig. S11). In agreement with a direct interaction, we observed DNA-induced chemical shift changes and signal attenuation. Both perturbations increased with increasing DNA concentration. Additionally, a specific signal intensity profile was observed at 17-fold excess of DNA over CD (*SI Appendix*, Fig. S11*A*). In agreement with strong CSPs at the N-terminus of the CD, these residues were attenuated by ~50%. The signal intensities then gradual rose toward residue 32 for which little signal attenuation was present. Downstream of residue 32, the signal intensities again decreased to reach a minimum of ~20% at residue E46, followed by an increase to about 60% for residues 52-72 and a subsequent increase to ~100% toward the C-terminus (*SI Appendix*, Fig. S11*A*). Notably, while DNA induced overall stronger signal attenuation in full-length HP1α, the residue-specific attenuation profile is highly similar for full-length HP1α and the isolated CD (Fig. 4 *D*, *Top* row and *SI Appendix*, Fig. S11*A*).

Having established a direct binding of the CD to DNA, we mapped the DNA-induced chemical shift perturbations onto the crystal structure of the CD. The analysis shows that the β-sheets as well as its C-terminal helix experience pronounced chemical shift changes (*SI Appendix*, Fig. S11*B*). The affected regions include the positively charged amino acids R29 and K40. Additionally, the negatively charged E46, E47, E52, E61, and D58 displayed CSPs and/or signal attenuation. Notably, glutamic and aspartic acid were found to be involved in sequence-specific DNA binding and readout (40). The specific binding of these acidic residues was suggested to arise from the balance between repulsion from negatively charged backbone phosphates and attractive interactions with cytosine (40).

## Discussion

The mechanism of HP1α-mediated formation of heterochromatin is still largely enigmatic (29, 41, 42). Recent studies suggested that cooperative liquid–liquid phase separation of HP1α and chromatin result in the compaction/association of chromatin and thus transcriptional inactivity (9–15). However, other molecular mechanisms not requiring liquid–liquid phase separation have also been suggested to guide the formation of heterochromatin such as HP1α-induced cross-linking of chromatin segments (32). Part of these different results/conclusions might be related to the difficulty to separate HP1α phase separation from chromatin compaction with experiments in cells or in vivo. Using carefully designed, highly controlled in vitro experiments, we here show that human HP1α can promote chromatin compaction/association at sub-phase separation concentrations, i.e., HP1α-mediated liquid–liquid phase separation is not essential for chromatin compaction/association.

The phase-separation ability of HP1 proteins was suggested to intimately relate to gene silencing (43). Existing hypotheses on how HP1α-mediated phase separation aids chromatin compaction and heterochromatin-mediated gene silencing include the seclusion of compacted chromatin into phase-separated HP1 droplets (10) as well as the exposure of buried nucleosomal regions that enhance the capabilities for multivalent interactions between nucleosomes (12). Moreover, histone methylation was suggested to trigger multivalent interactions between the methyl mark and the chromodomain of HP1 driving liquid–liquid phase separation which thus might regulate chromatin compartmentalization (13). Further regulation of HP1α phase separation was reported using peptides with different charge properties (43). In addition, DNA methylation was shown to restrict the growth of heterochromatin compartments (44). The liquid–liquid phase-separation hypothesis thus offers multiple opportunities for the versatile regulation of chromatin compaction.

However, liquid–liquid phase-separation was recently challenged as a major driver of chromatin compaction in differentiated cells, as the knockout of HP1α did not affect compaction (41, 45, 46). Instead, HP1α was suggested to bind and bridge H3K9me3-modified nucleosomes without inducing chromatin compaction (34, 41), whereby HP1α is supposed to provide an additional safeguard against spurious transcription activation (34). On the other hand, HP1α is required to establish heterochromatin clustering in embryonic cells (46). Differences in embryonic versus differentiated cells were attributed to a potentially dominant role of other HP1 paralogues or heterochromatin proteins in differentiated cells (46). HP1α might also have other activities, such as the maintenance of stable gene repression by mediating recognition and destruction of heterochromatic RNA transcripts (47).

Through the combined analysis of liquid–liquid phase separation and chromatin association of unmodified and H3K9me3 chromatin arrays at different concentrations, we demonstrate that HP1α-mediated compaction of H3K9me3 chromatin can occur in the absence of liquid–liquid phase separation (Figs. 2 and 3). This is consistent with observations of chromatin compaction in living mouse cells (34), but diverges from findings obtained using the *S. pombe* HP1 protein Swi6 (12). We attribute these differences to different amino acid sequences and structural properties of human Hp1α and *S. pombe* Swi6 (35). HP1α-mediated droplet formation may contribute to chromatin compaction, but such a potential contribution was small (~20%) in our assays (Fig. 2). In addition, in vitro droplet formation only occurred at high HP1α concentration (~30 μM) (*SI Appendix*, Fig. S2), which is well beyond the physiological concentration of HP1α (i.e., 1.0 ± 0.7 μM) (48). In contrast, chromatin association occurred at near-physiological concentrations of HP1α in the absence of phase separation. We however cannot exclude the possibility that LLPS in cells can occur at lower concentrations than those observed in our in vitro experiments, for example, due to molecular crowding or other unknown factors. Additionally, we currently do not know whether microphase separation (49, 50) may occur at physiological concentrations and contribute to chromatin compaction/association. Notably, H3K9me3 chromatin arrays underwent association by only ~50% in the presence of human HP1α, which may be related to the hypothesis that HP1α can bridge nucleosomes without inducing chromatin compaction/association (34, 41).

**Fig. 5.**
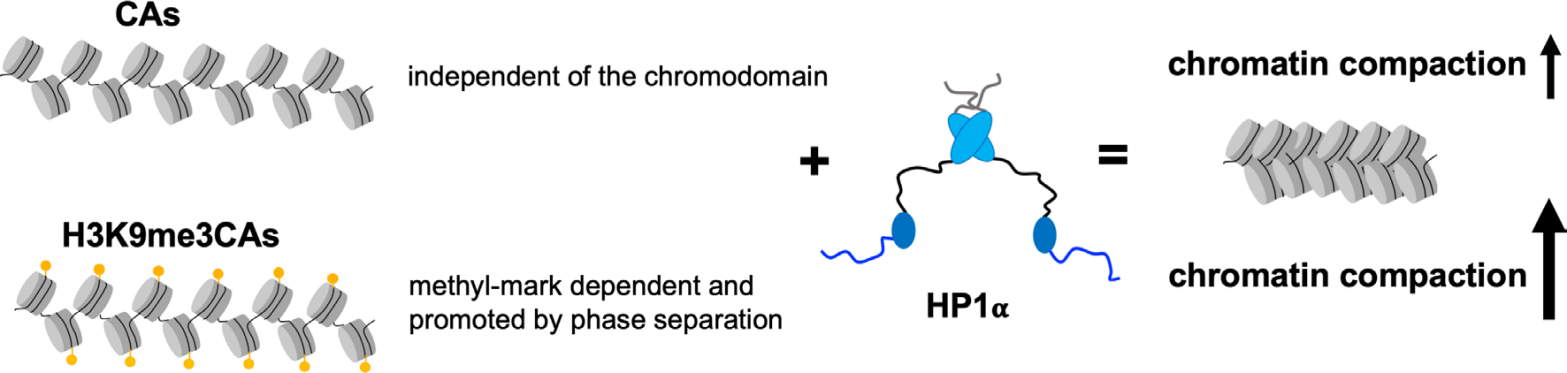
H3K9me3-dependent mechanism of HP1α-driven chromatin compaction. Schematic representation of the distinct HP1α-mediated compaction mechanisms of unmodified and H3K9me3 chromatin.

Our studies point to two distinct mechanisms of chromatin compaction, whose prevalence is regulated by chromatin H3K9me3 (Fig. 5). In the case of unmodified chromatin, the DNA-binding of the N-terminal disordered tail, the chromodomain, and the hinge region are important. Conversely, binding of the chromodomain to the methyl mark takes over a major role in the compaction of H3K9me3 chromatin, an interaction that is stabilized by the concurrent binding of the disordered N-terminal tail and hinge region presumably to inter-nucleosomal linker DNA. Two modes of interaction of HP1α with nucleosomes were suggested previously (51). In the first mode, interactions with linker DNA dominate, but when the linker DNA is missing or occluded by linker histones, HP1α might directly interact with the nucleosome core (51). Our findings extend the complexity of these two mechanisms demonstrating that the hinge region, the disordered N-terminal, and the chromodomain contribute to both mechanisms, albeit with different importance. Consistent with our findings, the disordered N-terminal tail of the *S. pombe* HP1 protein Swi6 was shown to participate, together with the hinge region and the chromodomain, in RNA binding as part of the capture of RNA transcripts (47). Our study further demonstrates that the chromodomain can directly bind to DNA, suggesting that the chromodomain binds competitively to histones and nucleosomal DNA. Additionally, competitive interactions between the chromodomain and the disordered N-terminal tail and hinge region for binding to DNA may occur. Notably, the possibility of chromodomain/DNA interaction was hypothesized in ref. 36 based on the observation of interactions between the chromodomain and RNA in fission yeast Chp1 and mammalian Cbx proteins (52). The importance of multiple protein and DNA interactions shown in the current work is further in agreement with single-molecule data suggesting the targeting of effectors to a specific chromatin modification state through multivalent interaction networks (53).

## Materials and Methods

### Plasmids.

The HP1α plasmid was kindly provided by Kurumizaka H. (54). Plasmids for the HP1α mutant proteins were produced by site-directed mutagenesis and confirmed by sequencing.

### Preparation of Recombinant HP1α.

HP1α (UniProt number P45973), the HP1α mutant proteins, and the CD of HP1α were expressed in *Escherichia coli* strain BL21 (DE3) from a pET15b vector. Unlabeled HP1α and mutant proteins were grown in 2l LB and induced with 0.5 mM IPTG. To obtain specifically labeled protein, cells were grown in LB, centrifuged, washed with M9 salts (Na_2_HPO_4_, KH_2_PO_4_, and NaCl), and resuspended in minimal medium (H_2_O) supplemented with the required source of labeling and induced with 0.5 mM IPTG followed Marley’s protocol (55). For backbone assignment experiments, we prepared ^15^N-^13^C labeled HP1α using ^15^NH_4_Cl and ^1^H, ^13^C-glucose in H_2_O-based minimal medium. Lysine labeled ^15^N-Lys HP1α was prepared by using ^15^N labeled lysine as the only source of lysine before inducing protein production by IPTG. After harvesting, the bacterial cells were resuspended in lysis buffer (50 mM Tris-HCl pH 7.5, 500 mM NaCl) complemented with protease inhibitor cocktail (cOmplete, Sigma-Aldrich), 0.4 mM MgCl_2_, 0.2 mM MnCl_2_, 0.2 mM CaCl_2_, lysozyme, and DNAse I and disrupted by sonication. Cell lysate was cleared by centrifugation, and the supernatant was filtered and applied to a HisTrap column (Cytiva). The column was washed with buffer A [50 mM Tris-HCl pH 7.5, 500 mM NaCl, 2 mM 2-mercaptoethanol (2-ME), and 5 mM imidazole], and the His-tagged proteins were eluted by a linear gradient from 5 to 500 mM imidazole. After overnight dialysis against 20 mM Tris-HCl pH 7.5, 300 mM NaCl, and 2 mM 2-ME, the NaCl concentration was further slowly diluted to 100 mM to avoid precipitation of the proteins. The His-tag of HP1α and mutant proteins was removed by PreScission Protease (Cytiva) and the samples were further purified by a MonoQ column (GE Healthcare) with a linear gradient from 100 to 1,000 mM NaCl. The His-tag of the CD of HP1α was removed by TEV cleavage at 34 °C for 4 h. The CD of HP1α was again applied to a HisTrap column and eluted by a linear gradient from 5 to 500 mM imidazole. The pH of the sample was adjusted to 5.0 and further purified by a MonoS column (GE Healthcare) equilibrated with 20 mM MES pH 6, 1 mM DTT, 0.1 mM PMSF, and 20 mM NaCl and eluted with a linear gradient from 100 to 1,000 mM NaCl. Fractions containing HP1α, HP1α mutant proteins, and CD of HP1α were additionally purified by a Superdex 75 column (GE Healthcare). The proteins were dialyzed against different buffers depending on the experiment and concentrated by centrifugation (5 kDa Vivaspin, Sartorius). Protein concentrations were determined by Nanodrop and flash-frozen aliquots were stored at −80 °C.

### Peptide Synthesis.

The HR peptide (Ac-NKRKSNFSNSADDIKSKKKREQS-NH2) and the HR peptide mutants were synthesized on a Libety1 (CEM) instrument. They were subsequently purified via HPLC (Reversed-phase, RP18, JASCO). Analysis was carried out by the liquid chromatography Acquity Arc system (WATERS with SQD2 single quadrupole mass detector).

### Solution Turbidity.

Turbidity values of protein-DNA samples were measured at RT and a wavelength of 500 nm using the NanoDrop spectrophotometer (ThermoFisher Scientific, Invitrogen). Average turbidity values are derived from three independent measurements.

### Protein and Peptide Acetylation.

The acetylation was carried out along the lines described in ref. 56. Specifically, HP1α was acetylated using CREB [recombinant hCREB binding protein (catalytic domain) from Enzo] and p300 [recombinant hp300 binding protein (catalytic domain) from Enzo]. The reaction was performed by mixing 50 μM of the protein with 0.62 μM CREB and 0.62 μM p300, 4 mM AcCoA, 0.5 mM PMSF, 0.1 mM EDTA, 2 mM DTT in 25 mM HEPES, pH 7.4. To acetylate the HR peptide, 1 mM of peptide was mixed with 0.62 μM CREB, 0.62 μM p300, 3 mM AcCoA, 0.5 mM PMSF, 0.1 mM EDTA, 1 mM DTT in 25 mM HEPES, pH 7.4. For efficient acetylation, samples were incubated at room temperature (RT) for 2 d under slow shaking conditions (300 rpm). Unbound AcCoA was removed by 700 MWKO Zeba spin desalting column (Thermo Fisher Scientific).

The sites and degree of acetylation were determined using NMR spectroscopy (*SI Appendix*, Fig. S3 *B* and *C*). Based on 2D ^1^H-^15^N correlation spectra of lysine-labeled HP1α, we identified four out of the 29 lysine residues to be acetylated: K3 and K6 in the NTE, K42 in the CD, and K91 in the hinge region (*SI Appendix*, Fig. S3*C*). The degree of acetylation of each of the four lysine residues was ~80%. Mass spectrometry confirmed onefold to fourfold acetylation of HP1α (*SI Appendix*, Fig. S3*D*). To further investigate the ability of acetyltransferases to acetylate lysine residues in the hinge region, we acetylated the HR peptide. According to mass spectrometry, both mono- and di-acetylated HR peptides were obtained. The 2D ^1^H-^15^N correlation spectra of the mono-acetylated HR peptide confirmed that K91 is preferably acetylated in the hinge region of HP1α (*SI Appendix*, Fig. S3*E*).

### Mass Spectrometry (MS).

The MS was performed as described in ref. 56. Specifically, mass spectra of acetylated and unmodified HP1α and HR peptide were acquired by liquid chromatography combined with mass spectrometry.

### Liquid–Liquid Phase Separation.

The liquid–liquid phase separation experiments of HP1α proteins and HR peptides with DNA (Salmon Sperm DNA, low molecular weight, by Sigma) and with chromatin arrays (CAs and H3K9me3CAs) were performed in 25 mM HEPES, pH 7.4 and, 10 mM Tris-HCl, pH 7.8, 75 mM KCl, 0.5 mM EDTA, respectively. HP1α protein experiments were carried out under reducing conditions at RT in the presence of 2 mM DTT or 1 mM TCEP to avoid cysteine oxidation.

### Labeling of Proteins with Fluorescent Dyes.

HR peptides were fluorescently labeled on the C-terminal end with fluorescein amidite (FAM). HP1α proteins were fluorescently labeled on the three native cysteine residues (C59, C133, and C160) using Alexa Fluor 488 C5 Maleimide dye (Thermo Fisher Scientific). The labeling was carried out as described in ref. 56. In particular, 15 moles of dye were used for labeling for each mole of the protein. Proteins were incubated for 2 h at room temperature in a light-protected Eppendorf tube with the dye freshly prepared in dimethyl sulfoxide (DMSO). The excess dye was removed by passing the sample twice through a 0.5-mL 700 MWKO Zeba spin desalting column (Thermo Fisher Scientific). AcHP1α was acetylated after labeling as described in the labeling section. Chromatin arrays, unmodified and H3K9me3, were stained with DAPI, and the excess dye was removed by passing the sample through a 0.5-mL 700 MWKO Zeba spin desalting column (Thermo Fisher Scientific).

### DIC and Fluorescence Microscopy.

DIC and fluorescence images were obtained on a Leica DM6000 B microscope with a 63× objective (water immersion) and 100× objective (oil immersion) at room temperature and processed with FIJI software (NIH) (57). For fluorescence imaging, FAM-labeled peptide/Alexa 488-labeled protein was added to the unlabeled protein/peptide samples.

### NMR Spectroscopy.

NMR spectra for backbone resonance assignment of HP1α backbone were acquired at 303 K on 900-MHz Brucker spectrometer equipped with a triple-resonance 5-mm cryogenic probe. The NMR sample contained 0.5 mM of HP1α in 20 mM MES (pH 6.2), 100 mM NaCl, 1 mM TCEP, and 10% D_2_O. The backbone resonances were assigned using the following NMR experiments: 2D ^1^H-^15^N TROSY-HSQC, 3D HNCO, 3D HNCA, 3D HNCOCA, 3D HNCACO, 3D CBCANH, and 3D CANNH. The recorded spectra were analyzed by CcpNmr Analysis version 2.4 (58). Likely due to oligomerization at high protein concentrations, most of the cross peaks of the CSD could not be assigned and the ^1^H-^15^N assignments of HP1α CSD were transferred from published assignments (39).

NMR spectra for the resonance assignment of the HP1α hinge region (HR) peptide were acquired at 278 K on a Bruker 950-MHz spectrometer equipped with a triple-resonance 5-mm cryogenic probe. The concentration of the HR peptide was 2 mM in 20 mM NaPB (pH 6.8). The HR peptide was assigned using two-dimensional ^1^H-^1^H TOCSY and ^1^H-^1^H NOESY experiments and supported by a natural abundance ^1^H-^15^N HMQC NMR spectrum. The NMR spectrum of the monoacetylated HP1α hinge region (HR) peptide was acquired at 278 K on a Brucker 800-MHz spectrometer. 2D natural abundance ^1^H-^15^N HMQC spectra were recorded at 0.7 mM concentration in 20 mM NaPB (pH 6.8). Chemical shifts were referenced to the chemical shifts of sodium 3-(trimethylsilyl)propane-1-sulfonate (DSS). Spectra were processed with TopSpin 3.6 (Bruker) and analyzed using Sparky (59).

NMR titrations of ^15^N-Lys hHP1α, ^13^C-^15^N labeled hHP1α, ^15^N labeled CD and ^15^N-Lys AcHP1α with DNA/chromatin were acquired at 303 K on a Bruker 800- and 900-MHz spectrometers. 2D ^1^H-^15^N HSQC and 2D ^1^H-^15^N TROSY-HSQC for ^15^N-Lys HP1α and ^13^C-^15^N hHP1α, respectively, were recorded for the proteins (50 μM) with increasing molar ratios of DNA (0.02 and 0.04) in 20 mM MES, pH 6.2, 100 mM NaCl, 1 mM TCEP and 10% D_2_O. Chemical shift perturbations (CSP) of individual residues were calculated according to CSP = √[(δ^1^H_bound_ − δ^1^H_free_)^2^ + (δ^15^N_bound_/10 − δ^15^N_free_/10)^2^], considering the relative dispersion of the proton and nitrogen δ chemical shifts.

### Nucleosome Preparation and Chromatin Array Assembly.

H3K9me3 was generated by native chemical ligation as described (33). Briefly, 0.2 mM of H3Δ1-20, A21C and 1 mM of N-terminal H3K9me3 peptide (1–20) with a C-terminal thioester group were incubated at room temperature for 40 h in 100 mM potassium phosphate, 3 M guanidine-HCl, 0.5% (v/v) benzyl mercaptan, 0.5% (v/v) thiophenol, pH 7.9 with vigorous mixing. The ligation reaction mixture was then dialyzed against SAU200 buffer overnight (7 M deionized urea, 20 mM sodium acetate pH 5.2, 1 mM EDTA, 1 mM DTT, 200 mM NaCl), and applied to a Hi-Trap SP-Sepharose high-performance cation exchange column and eluted with a linear NaCl gradient from 200 to 1,000 mM. Ligated protein fractions were pooled and dialyzed extensively against 2 mM DTT at 4 °C, lyophilized and stored at −80 °C.

In the next step, histone octamers were reconstituted. Shortly, lyophilized purified *Xenopus* core histones H2A, H2B, H3 and/or H3K9me3, H4 were dissolved in unfolding buffer (10 mM Tris-HCl, 7 M guanidinium hydrochloride, 10 mM DTT, pH 7.5) and mixed to equimolar ratios. The histone mixture was extensively dialyzed at 4 °C against RBH buffer (10 mM Tris-HCl, 1 mM EDTA, 2 M NaCl, 1 mM DTT, pH 7.5). Histone octamers were concentrated to 10–20 mg/mL using Amicon Ultra centrifugal filter units (Millipore, Billerica, USA) with a 30-kDa cutoff and purified on a Superdex 200 prep grade gel filtration column (GE Healthcare, Freiburg, Germany). Peak fractions were pooled and concentrated to at least 1–2 mg/mL. Histone octamers were stored in 50% (v/v) glycerol at −20 °C.

In the last step, oligonucleosomes were reconstituted on a 12 × 200 bp × 601 template (60). pUC18_12×200×601 plasmids carrying the 12-mer 601 inserts were purified using a Giga kit (Qiagen, Hilden, Germany). DNA templates for oligonucleosome reconstitution were released from plasmids by restriction digest with a mix of EcoRI, BanI, BfuCI, and HaeII restriction enzymes. To purify the digested 12 × 200 bp × 601 template from the vector backbone, stepwise precipitation with polyethylene glycol (PEG) 6000/0.5 M NaCl was performed [final PEG concentration 2 to 9% and 20% (w/v)]. DNA pellets were washed with 70% (v/v) ethanol, air dried, and dissolved in water. Histone octamers and DNA were mixed in RB high buffer (10 mM Tris-HCl, 2 M NaCl, 1 mM EDTA, 2 mM DTT, pH 7.5) at a molar ratio of 1.0–1.3. Using a peristaltic pump, a gradient to RB low buffer (10 mM Tris-HCl, 25 mM NaCl, 1 mM EDTA, 2 mM DTT, pH 7.5) was applied during dialysis at 4 °C over 36 h.

### Chromatin Association Assay.

The experiments were performed following the published assay (12) with some modifications. Chromatin self-association was carried out with 40 nM of chromatin arrays and 5 μM or 40 μM of HP1 proteins in 10 mM Tris-HCl, pH 7.8, 75 mM KCl, 0.5 mM EDTA and 2 mM DTT. After adding the HP1 proteins to the chromatin arrays, the samples were incubated for 30 min at 20 °C and centrifuged at 15,000 g for 10 min at 20 °C. The supernatants were transferred to a fresh tube and 2 μL was read at 260 nm using Nanodrop. For each experiment, three technical and two biological replicas were performed.

To calculate the array soluble fraction, we took as the reference (initial) chromatin value the value of chromatin upon subjecting the reference chromatin sample to the chromatin self-association assay. The assay itself reduced the chromatin concentration by 5 to 10%, which means that some of the chromatin was lost due to the assay. At the concentration 40 μM of HP1α, the protein also contributes to the absorption at 260 nm (at 5 μM, the protein absorption is negligible). The array soluble fraction was calculated by considering the loss of the chromatin during the assay as well as the absorption of the protein. The concentration of the protein not interacting with chromatin was determined from the supernatant using SDS Page gel analysis and considered in the calculations (*SI Appendix*, Fig. S12).

## Supplementary Material

Appendix 01 (PDF)

Dataset S01 (XLSX)

## Data Availability

The NMR assignments of HP1α are included in Dataset S1. All other data are included in the manuscript and/or supporting information.
