# Supplementary material for: Multimodal interactions drive chromatin phase separation and compaction
Source: Proc Natl Acad Sci U S A. 2023 Dec 4;120(50):e2308858120. doi: 10.1073/pnas.2308858120 (PMC10723116; doi:10.1073/pnas.2308858120)
Supplement: Supplementary file 1 — Appendix 01 (PDF) [file pnas.2308858120.sapp.pdf]

Supplementary Information

Multimodal interactions drive chromatin phase separation and compaction

Tina Ukmar-Godec[1], Maria-Sol Cima-Omori[1], Zhadyra Yerkesh[2], Karthik Eswara[2], Taekyung Yu[1], Reshma Ramesh[1], Gwladys Riviere[1], Alain Ibanez de Opakua[1], Wolfgang Fischle[2], and Markus Zweckstetter[1,4*]

[1] German Center for Neurodegenerative Diseases (DZNE), Translational Structural Biology, Von-Siebold-Str. 3a, 37075 Göttingen.

[2] Bioscience Program, Biological and Environmental Science and Engineering Division, Laboratory of Chromatin Biochemistry, King Abdullah University of Science and Technology (KAUST), Thuwal 23955, Saudi Arabia.

[3] Max Planck Institute for Multidisciplinary Sciences, Department of NMR-based Structural Biology, Am Fassberg 11, 37077 Göttingen, Germany.

[*] correspondence: Markus.Zweckstetter@dzne.de

Supplementary Figures

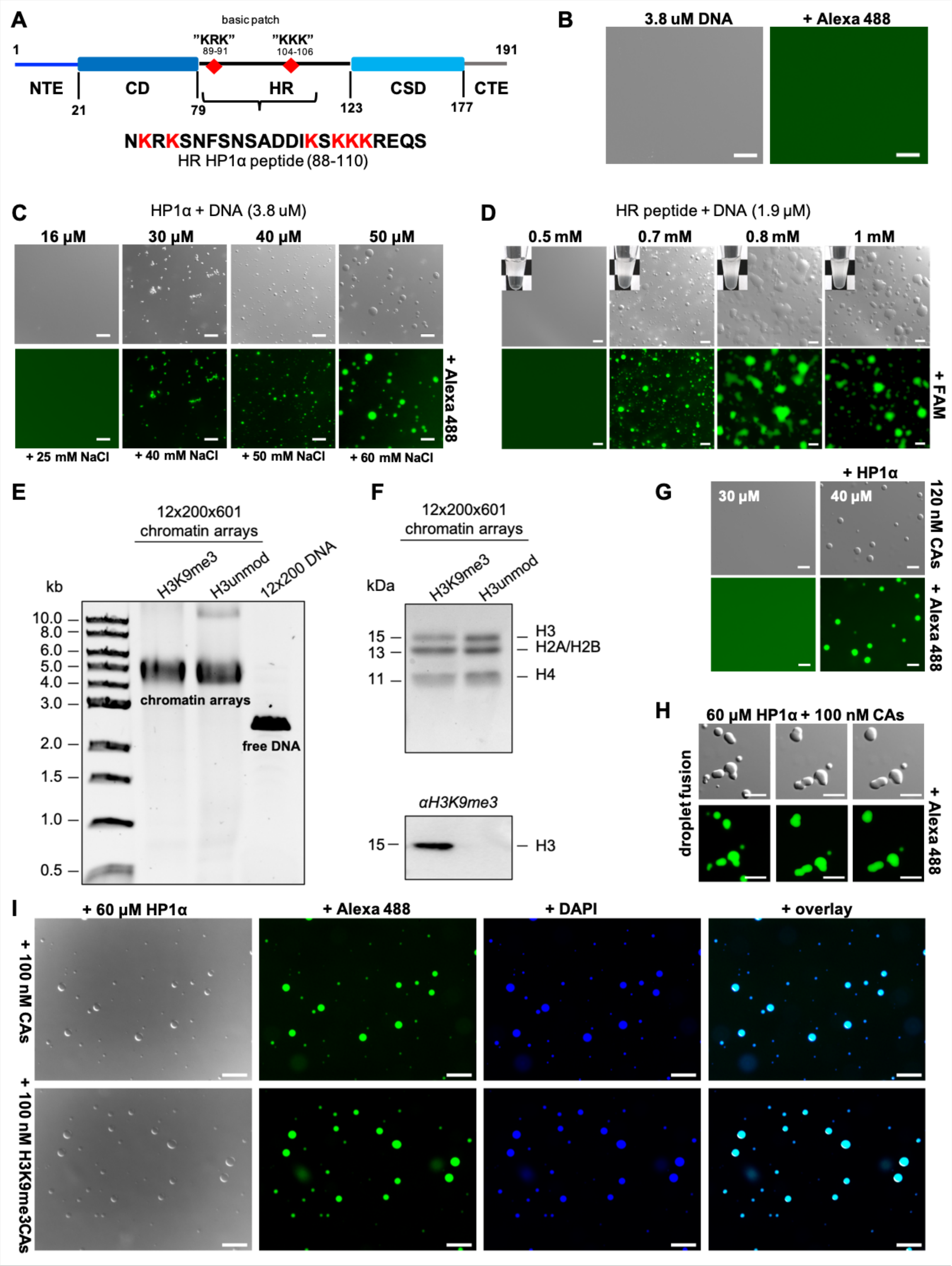

Fig. S1. Concentration-dependent liquid-liquid phase separation of HP1α and HR peptide. (**A**) Schematic representation of the amino acid sequence of HP1α. CD and CSD are the chromo and chromo-shadow domain, respectively, NTE/CTE the N/C-terminal disordered tails, and HR the disordered hinge region. The amino acid sequence of the HR peptide is shown below; lysine residues are marked in red. (**B**) DIC and fluorescence images of mixtures with DNA at concentrations where DNA does not phase separate by itself; these concentrations were used in liquid-liquid phase separation experiments of HP1α and HR peptide in the presence of DNA. (**C**) DIC and fluorescence images of mixtures of HP1α with DNA in 25 mM HEPES, pH 7.4, 2 mM DTT and same molar

protein/salt ratio to ensure the same screening and thus strength of electrostatic interactions. (**D**) DIC and fluorescence images of mixtures of HR peptide with DNA 25 mM HEPES, pH 7.4 and 2 mM DTT. Photographs as insets demonstrate increasing solution turbidity at higher HR peptide concentrations. (**E**) Native agarose gel electrophoresis followed by ethidium bromide staining of 12x200x601 chromatin (CAs) arrays. Free 12x200x601 DNA is included as a control. (**F**) SDS-PAGE analysis of H3K9me3 and unmodified histone H3, 12x200x601 chromatin (CAs) arrays stained with Coomassie blue (top). The presence of the modified H3K9 mark was verified by western blotting against H3K9me3 (bottom). Molecular weights of histone proteins are indicated (left). (**G**) DIC and fluorescence images of mixtures of HP1α with unmodified CAs at the higher concentration of 120 nM. (**H**) DIC and fluorescence images of fusing HP1α/CA droplets. For fluorescence imaging, unlabeled peptide/protein samples were mixed with FAM-labeled peptide (1 μM) or Alexa 488-labeled protein (0.6 μM). (**I**) DIC and fluorescence images of mixtures of HP1α with unmodified and H3K9me3 CAs. For fluorescence imaging, the unlabeled protein samples were mixed with Alexa 488-labeled protein (0.6 μM) and chromatin arrays were stained with DAPI. Buffer used in (**G** to **I**): 10 mM Tris-HCl, pH 7.8, 75 mM KCl, 0.5 mM EDTA, 1 mM TCEP. Scale bars, 10 μm.

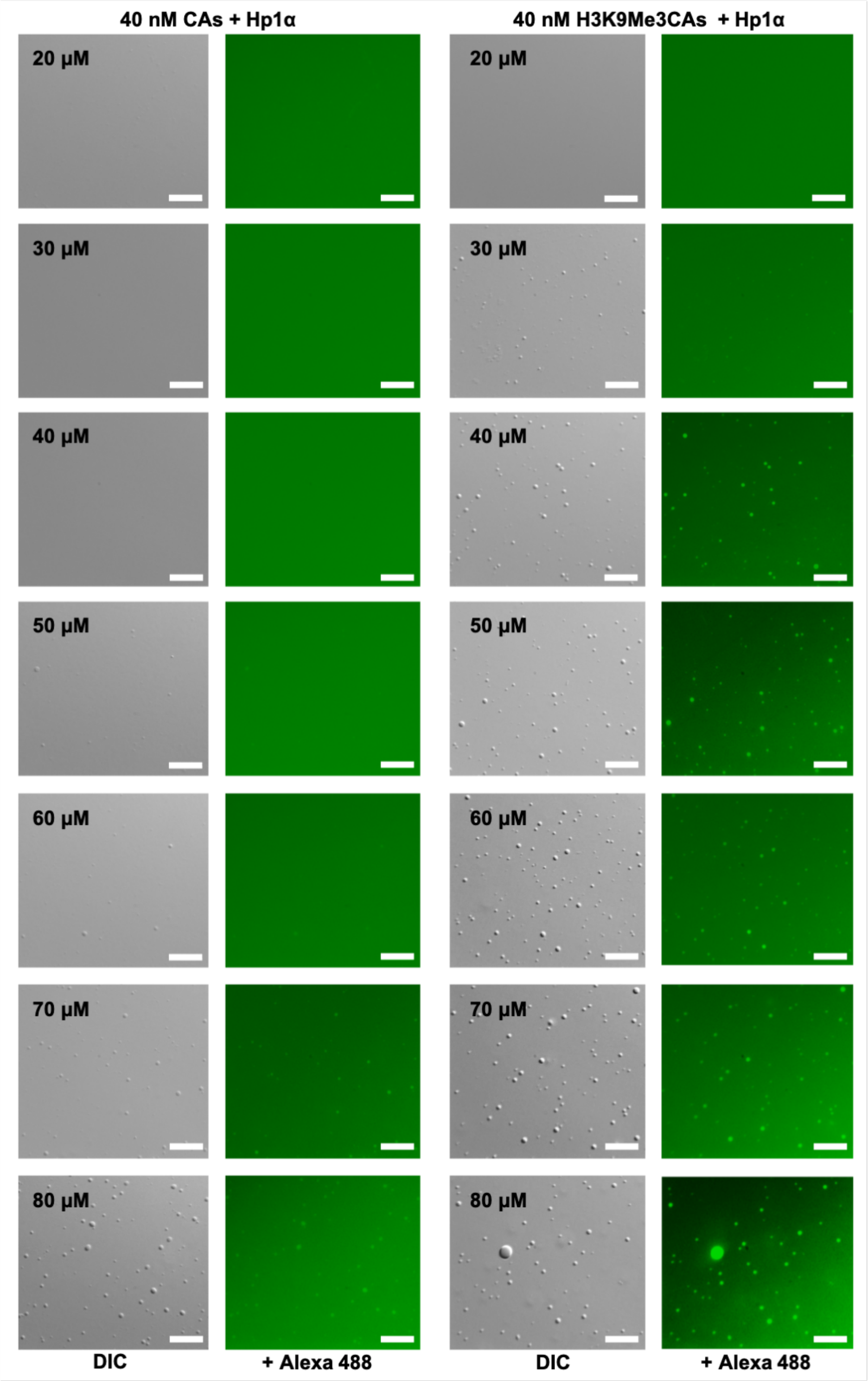

Fig. S2. Concentration-dependent liquid-liquid phase separation of HP1α by CAs and H3K9me3CAs. DIC and fluorescence images of samples at increasing concentrations of HP1α in the presence of unmodified CAs, **left panel** and in presence of H3K9me3CAs, **right panel** in 10 mM Tris-HCl, pH 7.8, 75 mM KCl, 0.5 mM EDTA, 1 mM TCEP buffer. Scale bars, 10 µm.

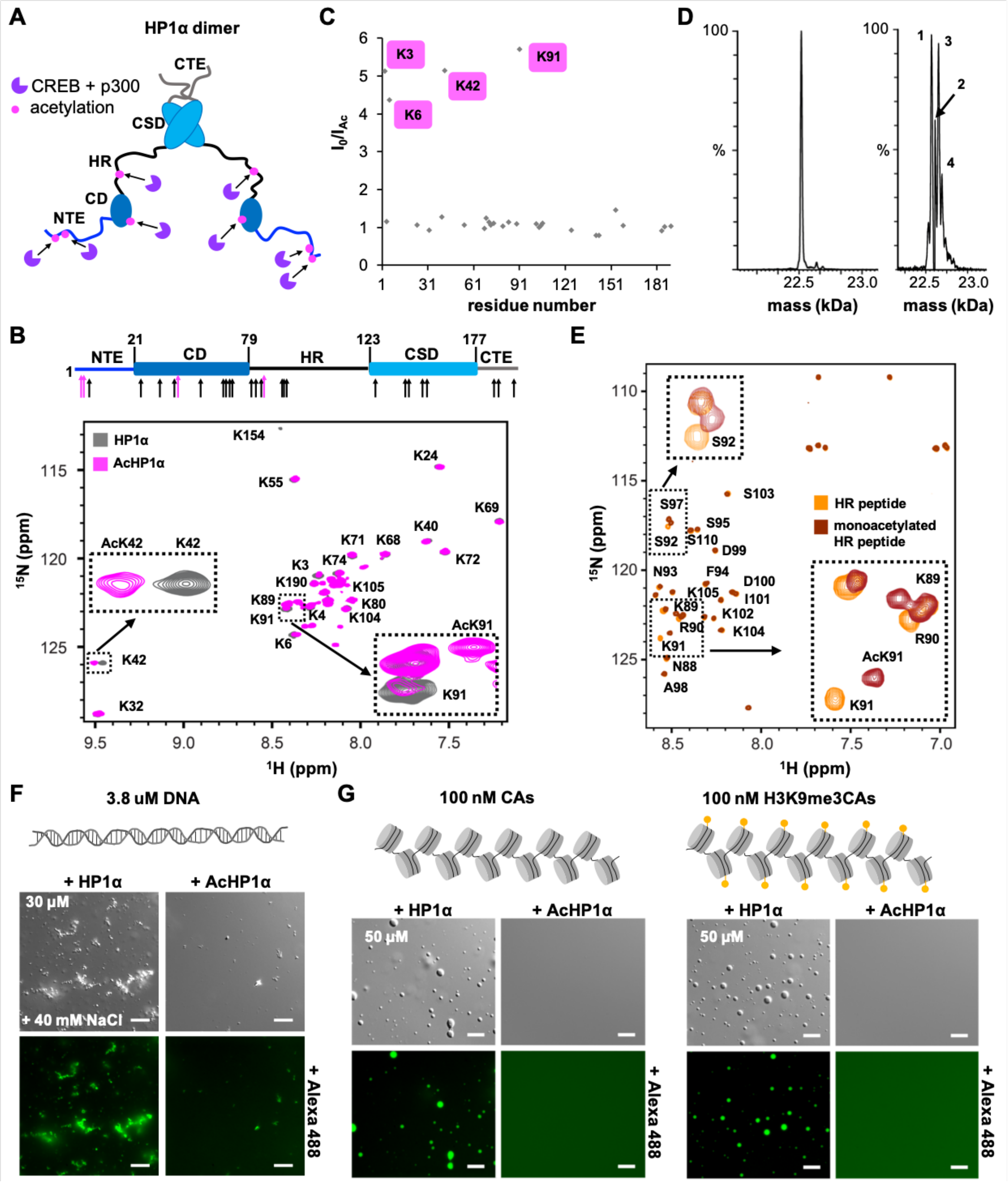

Fig. S3. **HP1α acetylation suppresses chromatin array phase separation.** (**A**) Schematics showing HP1α acetylation by two acetylases (p300 and CREB) of the four lysine residues located in the NTE, CD and HR. (**B**) 2D ^{1}H–^{15}N HSQC NMR spectrum of ^{15}N-lysine labelled HP1α showing the superposition of unmodified HP1α (grey) and acetylated HP1α (magenta). The dotted boxes show the perturbation of chemical shifts upon acetylation of lysine K42 and K91. Domain organization of HP1α depicting the location of the four acetylated lysine residues (arrows in magenta) is displayed above. (**C**) Residue-specific peak intensity ratios observed between unmodified HP1α and AcHP1α in 2D ^{1}H–^{15}N HSQC spectra; the four lysine residues that become acetylated are highlighted. (**D**) Mass spectra of HP1α (left) and AcHP1α after acetylation by p300 and CREB (right). (**E**) Superposition of 2D ^{1}H–^{15}N HMQC spectra of HR peptide and monoacetylated HR peptide; insets: enlargement of chemical shift perturbations of K91 and S92 as the residue next to K91. (**F**) DIC and fluorescence images of mixtures of unmodified HP1α (left) and AcHP1α (right) with DNA in 25 mM HEPES, pH 7.4 and 2 mM DTT. (**G**) Comparison

of DIC and fluorescence images of mixtures of HP1α and AcHP1α with CAs (left) and H3K9me3CAs (right) in 10 mM Tris-HCl, pH 7.8, 75 mM KCl, 0.5 mM EDTA, 1 mM TCEP buffer. For fluorescence imaging, unlabeled HP1α samples were mixed with Alexa 488-labeled protein (0.6 µM). Scale bars, 10 µm.

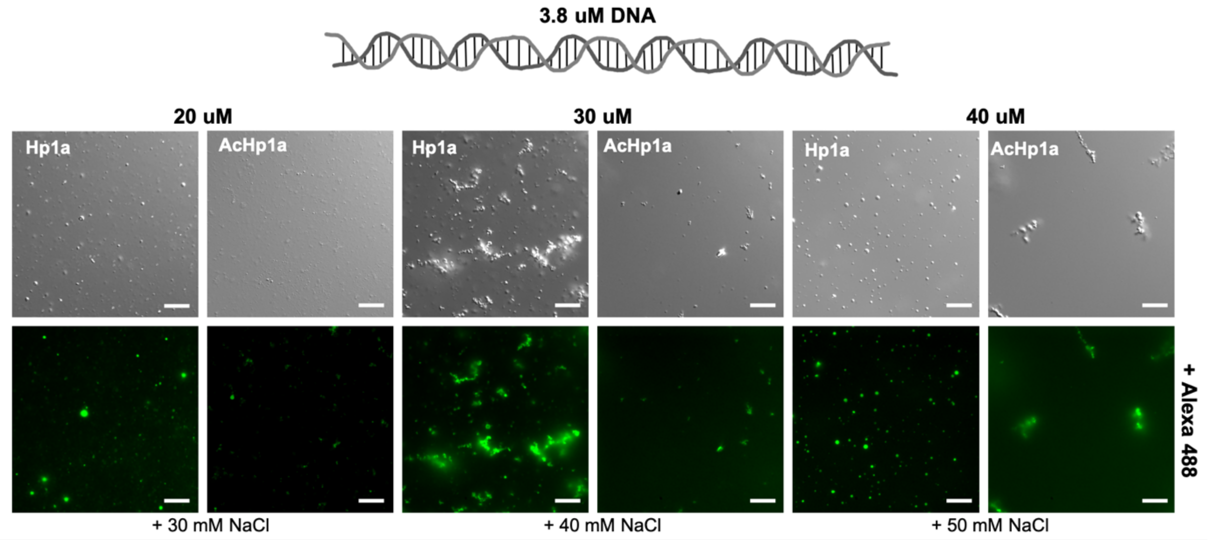

Fig. S4. Concentration-dependent liquid-liquid phase separation of HP1α in presence of DNA. DIC and fluorescence images of mixtures of unmodified HP1α and AcHP1α with DNA in 25 mM HEPES, pH 7.4 and 2 mM DTT. Scale bars, 10 µm.

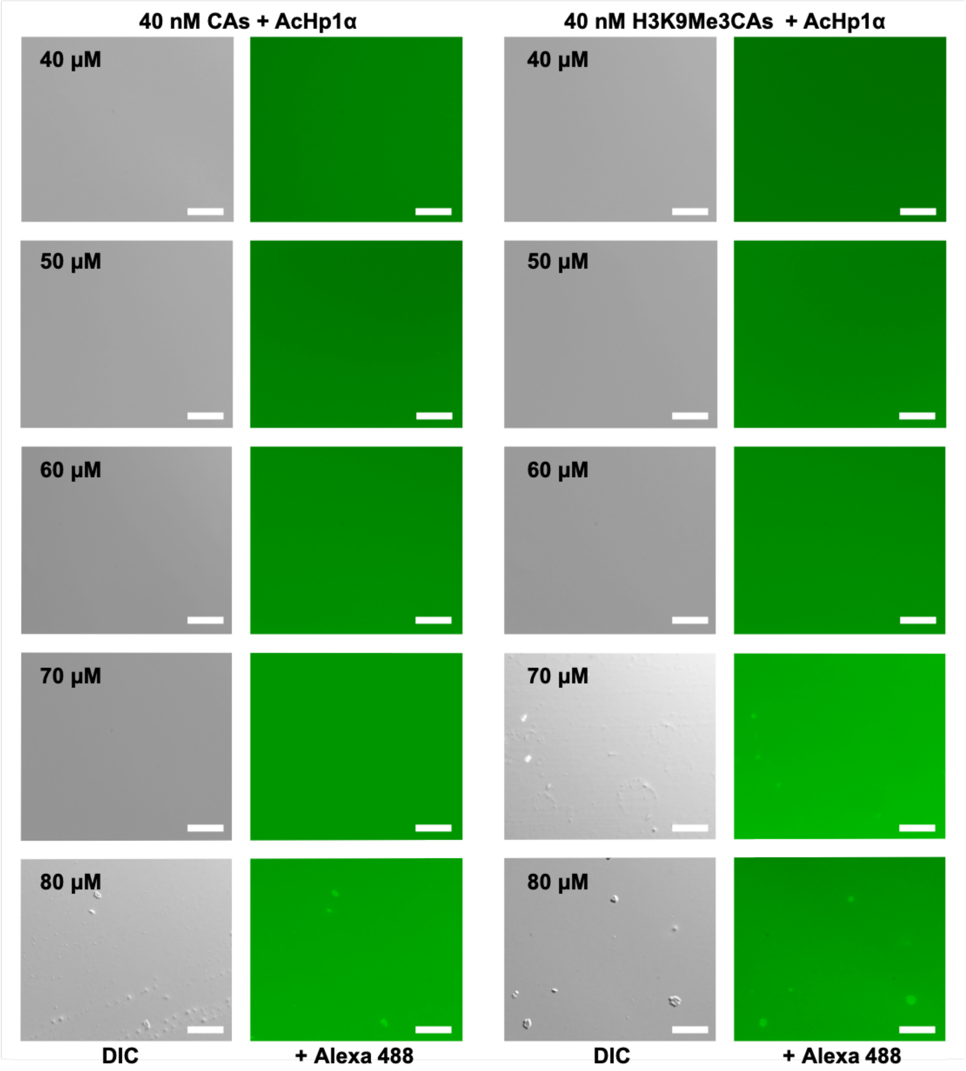

Fig. S5. Concentration-dependent liquid-liquid phase separation of AcHP1α by CAs and H3K9me3CAs. DIC and fluorescence images of increasing concentrations of AcHP1α in the presence of unmodified CAs, **left panel** and in presence of H3K9me3CAs, **right panel** in 10 mM Tris-HCl, pH 7.8, 75 mM KCl, 0.5 mM EDTA, 1 mM TCEP buffer. Scale bars, 10 µm.

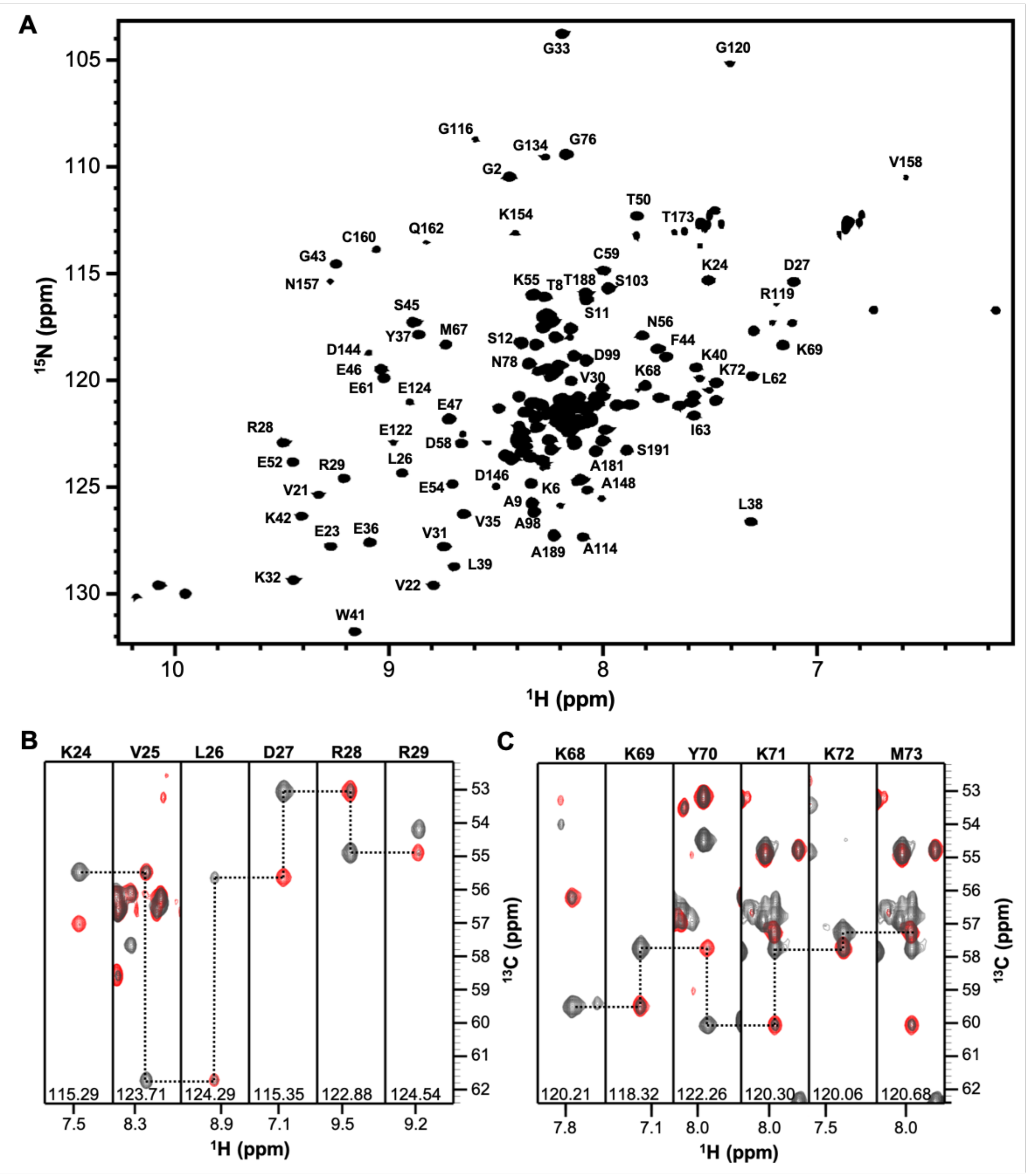

Fig. S6. **Resonance Assignment of human HP1α.** (**A**) 2D ^{1}H–^{15}N TROSY-HSQC NMR spectrum of full-length HP1α. (**B to C**) Strips of sequential resonance assignment from 3D HNCA (black) and 3D HNCOCA (red) for K24, V25, L26, D27, R28 and R29 (**B**), and for K68, K69, Y70, K71, K72 and M73 (**C**). The connectivity between Cαi and Cαi-1 is shown by dashed lines. Note that some assignments of the residues in the hinge region are missing because they could not be unambiguously assigned (please see Dataset S1 of the HP1α assignments).

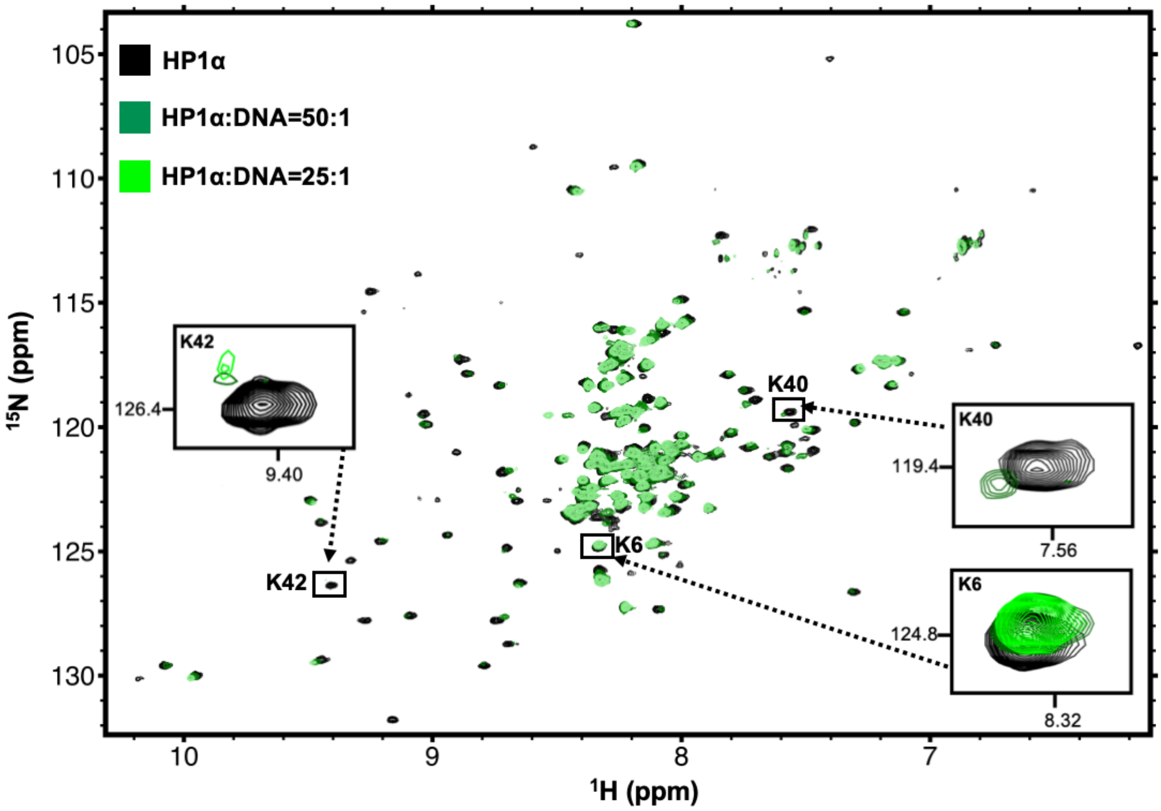

Fig. S7. **NMR spectroscopy of the interaction of Hp1α with DNA.** Superposition of 2D ^{1}H–^{15}N HSQC spectra of ^{15}N-labelled HP1α (black) without DNA or in the presence of HP1α:DNA molar ratios of 50:1 (dark green) and 25:1 (green); the cross-peaks of K6 (NTE), K40 (CD) and K42 (CD) are highlighted.

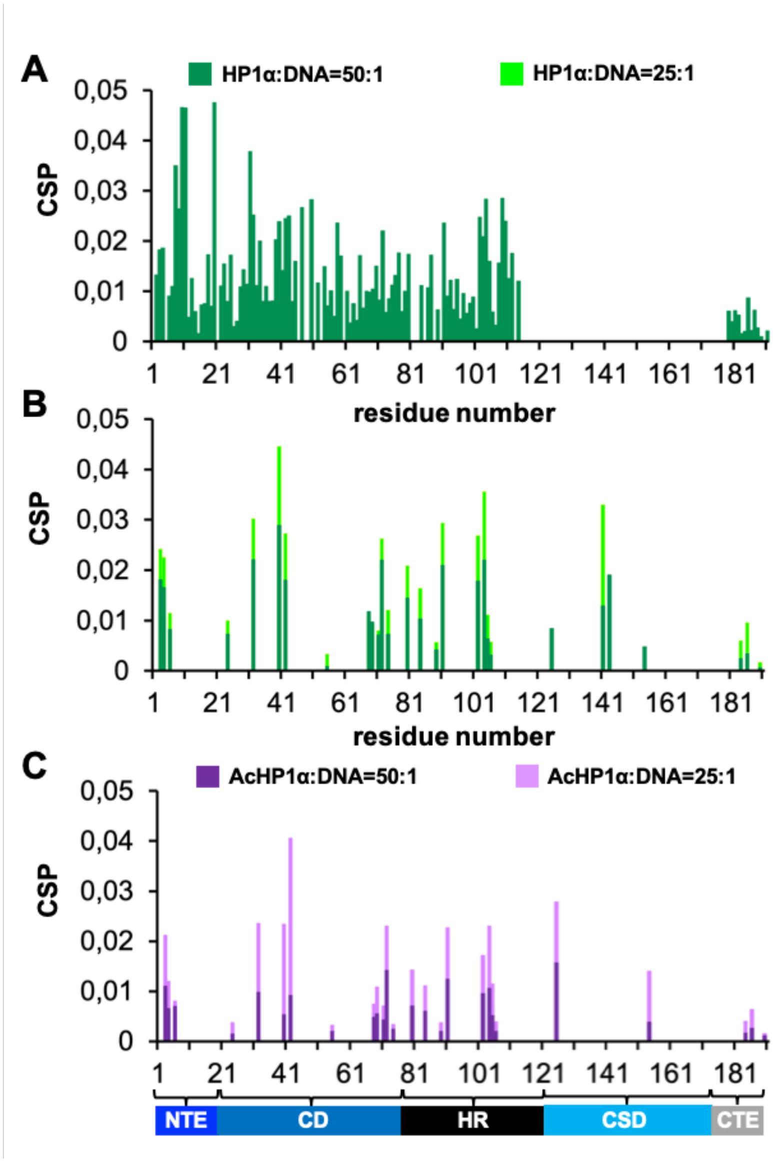

Fig. S8. **DNA-induced chemical shift perturbations in Hp1α and AcHp1α.** Averaged ^{1}H,^{15}N chemical shift perturbations (CSP) of resonances in 2D ^{1}H-^{15}N HSQC spectra of uniformly ^{15}N-labelled HP1α (**A**), ^{15}N-lysine labelled HP1α (**B**) and ^{15}N-lysine labelled AcHP1α (**C**) at increasing HP1α:DNA molar ratios (HP1α or AcHp1α /DNA molar ratio 50:1 (dark green or purple) and 25:1 (green or lila)). The domain organization of HP1α is shown below.

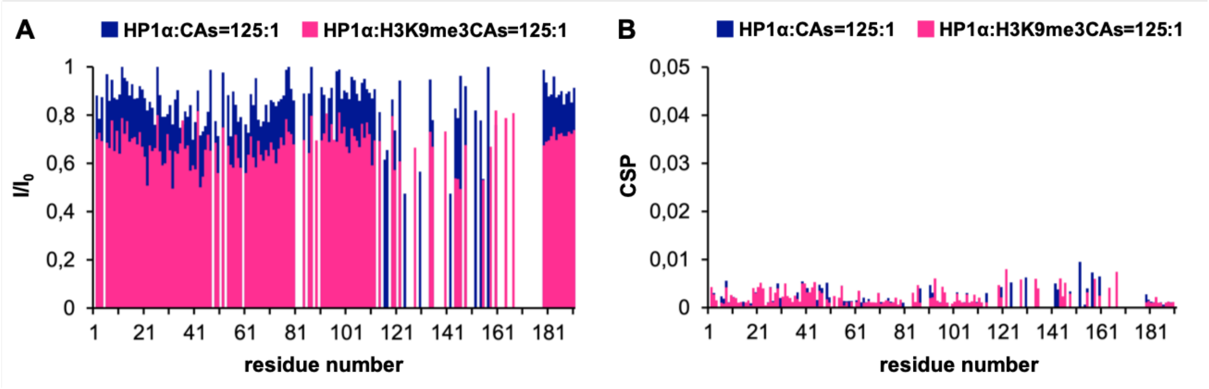

Fig. S9. NMR spectroscopy of the interaction of Hp1α with unmodified and H3K9me3 chromatin. Superposition of relative peak intensities (**A**) and averaged $^{1}H,^{15}N$ chemical shift perturbations (CSP) of resonances in 2D ^{1}H-^{15}N HSQC spectra (**B**) of ^{15}N-labelled HP1α for HP1α:CAs (dark blue) and HP1α:H3K9me3CAs (dark pink) molar ratio of 125:1. Note that the ratio of HP1α to binding sites alone is in fact much smaller, as there are two H3 tails per nucleosome and there are 12 nucleosomes on each array. Thus, the effective ratio HP1α to chromatin binding sites is approximately 5:1.

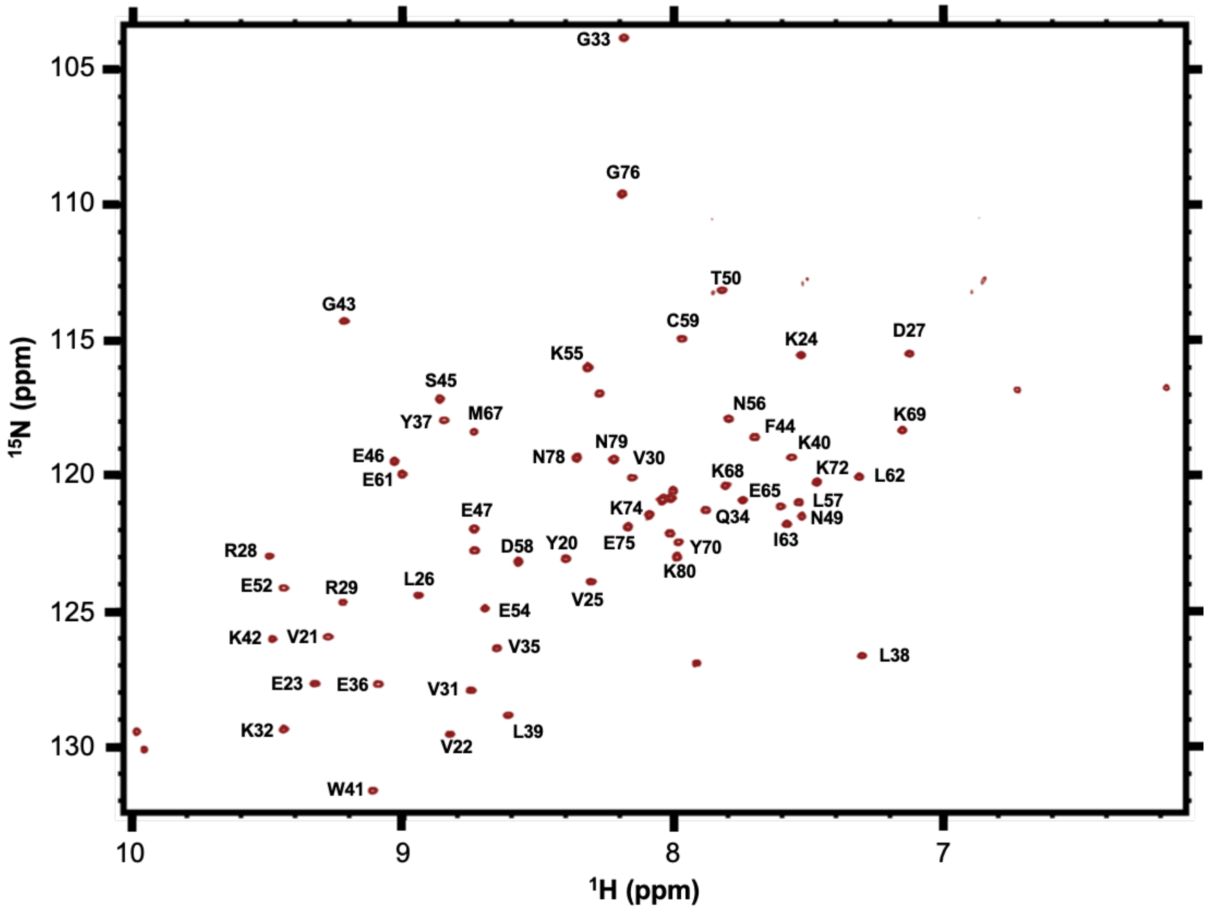

Fig. S10. **Resonance assignment of the isolated chromodomain of human HP1α.** (**A**) 2D ^{1}H–^{15}N TROSY-HSQC NMR spectrum of ^{15}N-labelled chromodomain (CD) of HP1α.

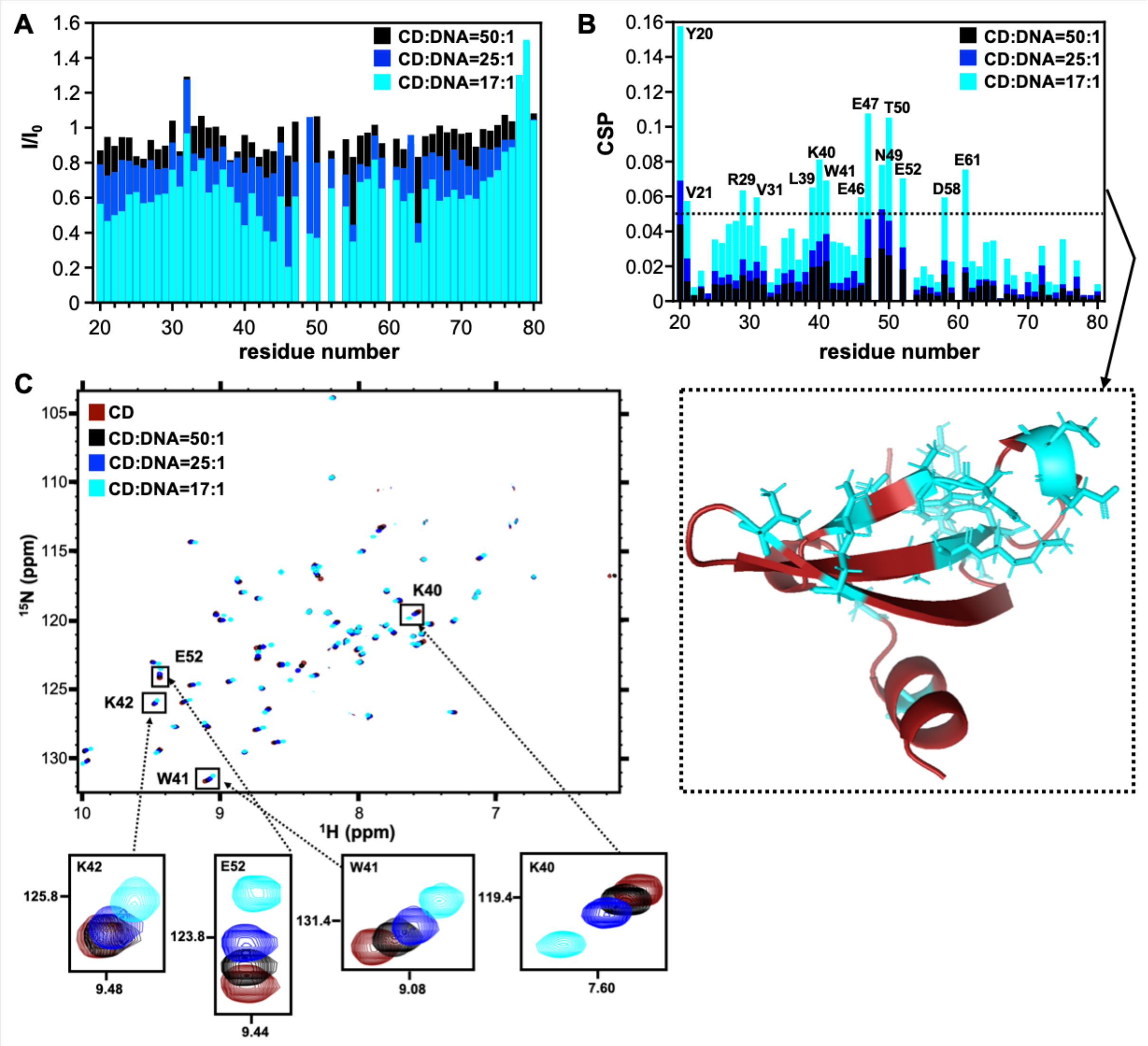

Fig. S11. **NMR spectroscopy of the interaction of the chromodomain (CD) of Hp1α with DNA.** (**A**) Relative peak intensities of ^{15}N-labelled CD of HP1α at CD:DNA molar ratios of 50:1 (dark red), 25:1 (dark blue) and 17:1 (cyan). (**B**) Averaged ^{1}H,^{15}N chemical shift perturbations (CSP) of resonances in 2D ^{1}H-^{15}N HSQC spectra of ^{15}N-labelled CD of HP1α at different CD:DNA (molar ratios and color code as in **A**). The dotted box shows the structure of the CD of HP1α (PDB code: 3fdt) with the amino acids that are involved in the binding to DNA and are represented as sticks in cyan (visualized with PyMOL Molecular Graphics System version 2.1.). The CSP cutoff is 0.05. (**C**) Superposition of 2D ^{1}H–^{15}N HSQC spectra of the CD of HP1α (dark red) without DNA or in the presence of CD:DNA molar ratios of 50:1 (black), 25:1 (dark blue) and 17:1 (cyan); the superposition of selected cross-peaks of 2D ^{1}H–^{15}N HSQC spectra of CD for increasing molar ratios of DNA of K40, W41, K42 and E52 are highlighted below the spectrum.

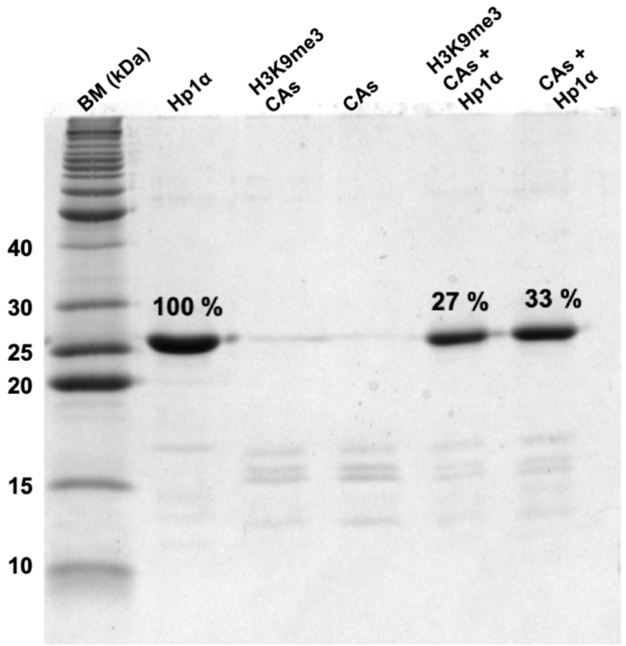

Fig. S12. Quantification of Hp1α not involved in chromatin association. SDS-PAGE gel of Hp1α, chromatin samples and the supernatant after chromatin association assay. The percentage of the protein in the supernatant was estimated using ImageJ. The percentage was taken into account when calculating the degree of chromatin association at 40 μM of Hp1α.
